# New Colors for Histology: Optimized Bivariate Color Maps Increase Perceptual Contrast in Histological Images

**DOI:** 10.1371/journal.pone.0145572

**Published:** 2015-12-30

**Authors:** Jakob Nikolas Kather, Cleo-Aron Weis, Alexander Marx, Alexander K. Schuster, Lothar R. Schad, Frank Gerrit Zöllner

**Affiliations:** 1 Institute of Pathology, University Medical Center Mannheim, University of Heidelberg, Mannheim, Germany; 2 Computer Assisted Clinical Medicine, Medical Faculty Mannheim, University of Heidelberg, Mannheim, Germany; 3 Department of Ophthalmology, Mainz University Medical Center, Mainz, Germany; Glasgow University, UNITED KINGDOM

## Abstract

**Background:**

Accurate evaluation of immunostained histological images is required for reproducible research in many different areas and forms the basis of many clinical decisions. The quality and efficiency of histopathological evaluation is limited by the information content of a histological image, which is primarily encoded as perceivable contrast differences between objects in the image. However, the colors of chromogen and counterstain used for histological samples are not always optimally distinguishable, even under optimal conditions.

**Methods and Results:**

In this study, we present a method to extract the bivariate color map inherent in a given histological image and to retrospectively optimize this color map. We use a novel, unsupervised approach based on color deconvolution and principal component analysis to show that the commonly used blue and brown color hues in Hematoxylin—3,3’-Diaminobenzidine (DAB) images are poorly suited for human observers. We then demonstrate that it is possible to construct improved color maps according to objective criteria and that these color maps can be used to digitally re-stain histological images.

**Validation:**

To validate whether this procedure improves distinguishability of objects and background in histological images, we re-stain phantom images and N = 596 large histological images of immunostained samples of human solid tumors. We show that perceptual contrast is improved by a factor of 2.56 in phantom images and up to a factor of 2.17 in sets of histological tumor images.

**Context:**

Thus, we provide an objective and reliable approach to measure object distinguishability in a given histological image and to maximize visual information available to a human observer. This method could easily be incorporated in digital pathology image viewing systems to improve accuracy and efficiency in research and diagnostics.

## Introduction

### Visual perception of histological images

The diagnosis of many, especially of malignant, diseases, relies on the evaluation of histological sections by a pathologist. Also, in basic and translational research, interpretation of histological sections is the basis for scientific conclusions. Accuracy and efficiency of this visual diagnostic process is determined by a number of factors. Four of these factors are commonly considered: first, the original tissue sample must comply with certain standards, then, the fixation and staining procedures should be standardized and optimized [1–4]. Third, the optical properties of the diagnostic microscope or digital scanner, the characteristics of the image acquisition (e.g. CCD-chip) and data storage should be calibrated to achieve capture a maximum amount of information present in the sample [5, 6]. Lastly, for digital pathology, display calibration can have an effect on diagnostic efficacy [7]. However, there is an additional information bottleneck in the diagnostic cascade of tissue samples: in order to be interpreted, the histological image information has to be processed by the visual system of the evaluating pathologist [8, 9]. Even if all sample preparation steps are fully optimized, it is conceivable that the resulting image is not optimally suited for the visual system of an observer and that therefore, a proportion of the available information is lost.

### Information transfer through the human visual system

The actual information that has to pass the visual system of the human observer is encoded as distinct—i.e. visually distinguishable—objects in the images perceived against a background. More generally, both inter-class-difference (i.e. contrast of objects against background) and intra-class-difference (i.e. contrast between different objects or even within a given object) are the actual basic entities of the encoded information. In a very simple view, these perceivable differences between objects are *intensity* differences and dark objects on a bright background are optimally distinguishable. However, the human visual system can differentiate approximately 10 million *colors* [10], as opposed to 720 gray scale intensity steps [11]. It follows that *color differences* are a much more powerful carrier of visual information than mere *intensity differences*.

### Image optimization in medical imaging

Some diagnostic imaging modalities such as computed tomography (CT) or magnetic resonance (MR) imaging produce data that is commonly encoded as intensity differences [11]. Just in the last few years, there have been structured approaches of digitally coloring radiological images to enhance diagnostic accuracy [12–15]. In contrast, histopathology has always offered a wider channel for transmission of visual information because images are *colored* due to the stain dyes. Yet, in most histopathological settings, the effectiveness of visual information transmission by different color hues is not actively measured or optimized. For example, the blue–brown colors in Hematoxylin—DAB (H–DAB) double-staining (a commonly used immunohistochemical (IHC) method) are not derived from considerations about the human visual system. Instead, these colors result from the chemical composition of the used dyes that are optimized for their binding properties. Based on our experience with histopathological images, we hypothesized that perceptual contrast in H–DAB images are not optimal for human visual perception, i.e. that the stain dye colors do not optimally fit the properties of the human visual system. Therefore, in the present study, we cast a closer look on the colors histological images are composed of and strived to find a way to retrospectively optimize these colors without chemical modifications of the stain dyes.

### Color optimization methods in digital pathology

Some authors have presented methods to retrospectively optimize the colors of histological, Hematoxylin & Eosin (H&E)—stained images. For example, Khan et al. combined a variant of color deconvolution with mapping to a new color space to achieve a standardized aspect of different histological images [16]. Similarly, Bautista et al. recently presented a method to optimize H&E—stained images to achieve a comparable color scheme in different images [17]. Another image optimization approach has been presented by Reyes-Aldasoro, who suggested an approach to correct unequal shading in a given image [18]. However, while in these studies, the authors optimized the red—blue color scheme of H&E—stained images, they still restricted the resulting colors to the original color hues, e.g. red and blue. Also, in digital pathology systems, overall image contrast, brightness and gamma of the resulting images is optimized, but the color hues themselves are not [7]. One study tested whether changing the hues of H&E—stained images could make these images more aesthetically pleasing for observers with impaired color vision [19]. However, in this study, the authors did not systematically investigate whether a new color scheme changes perceptual contrast or which color choices are optimal.

To the best of our knowledge, it has never been investigated whether by optimizing the colors of a given image, this image could yield more visually perceivable information to a human observer. Furthermore, little is known about color optimization in Hematoxylin (H)—3,3’-Diaminobenzidine (DAB) IHC images, although these images are important for qualitative and quantitative evaluation of tissue samples in a number of diseases, especially solid tumors [4].

### Aim of this study

In the present study, we strived to close a conceptual gap in color optimization of histological images. We investigated whether perceptual contrast of different objects in H–DAB immunostained images could be enhanced by optimally transforming the color scheme of a given image. In particular, we investigated which combination of hues are optimally suited for this task, and how the perceptual contrast could be optimized for these hues.

To answer these questions, we applied and validated self-developed methods for optimized stain unmixing, for measuring the bivariate color map inherent in a given histological image and replacing it with a better color map. Our aim was to examine properties of native color maps by a fully objective and reproducible approach and likewise examine the properties of perceptually improved color maps. In particular, we strived to find an optimal solution for color selection in immunostained histological images by using optimization methods for digital re-staining.

## Materials and Methods

### Ethics Statement

In this study, we used anonymized images of tissue samples of patients. One sample image is derived from our previous publication [20]. One image is a Creative Commons image (see List B in S1 File). Three H&E sample images derived from our local pathology archive and were already fully anonymized at the time of the experiments. All other images are published in the ProteinAtlas database (see http://www.proteinatlas.org/about/datausage).

### Digital re-staining of histological images

IHC images can be thought of as bivariate datasets: In each image pixel, the affinity of the antibody and the counterstain dye is measured (both are non-linear processes). Then, both measurements are combined to a color information at this given pixel. All possibly achievable colors in a given image are part of the set of available colors, which can be thought of as a bivariate color map. In native images, this bivariate color map is determined by the light absorption properties of the color pigment. Normally, an observer can only see the resulting image but does not know the exact bivariate color map the image is based on. In the present study, we developed a novel method to extract the color map that is inherent in a given image and replace them with an optimized set of colors (i.e. a new color map). To this end, we developed, employed and validated a novel method of several successive steps:

1. Retrieve an immunostained image as an RGB file (no further knowledge about the image is needed).
2. Extract the intensity information for the two stain channels by using an optimized variant of color deconvolution.
3. Use intensity information from both channels to extract the original color map. Missing parts are linearly interpolated.
4. Create an optimized color map of identical size as the original color map. Unlike the original color map, the new color map is perceptually linear.
5. For each item in the old color map, extract the image pixels that use this color value and replace their color with the corresponding item from the new color map.

The exact methods used in these steps will be explained in the following paragraphs.

### Optimized unsupervised color deconvolution

To extract the intensity information for each stain color separately, we developed and validated an optimized variant of Ruifrok’s color deconvolution [21]. In their original publication, Ruifrok et al. propose a standard matrix *M*_*Rui*_ for Hematoxylin—DAB—Eosin stainings. This standard matrix is widely used, e.g. for H–DAB staining and yields reasonable results for stain separation of three-channel and two-channel images. *M*_*Rui*_ is a 3 by 3 matrix and its row vectors are the basis vectors for three color channels (or two channels and a residual). In two-channel IHC images, the row vectors of *M*_*Rui*_ are ${\vec{H}}_{Rui}$ (for Hematoxylin), ${\vec{D}}_{Rui}$ (for DAB) and ${\vec{\epsilon }}_{Rui}$ (for the residual). The widely used ImageJ/Fiji software includes a scaled version of Ruifrok’s standard values.

**Fig 1:**
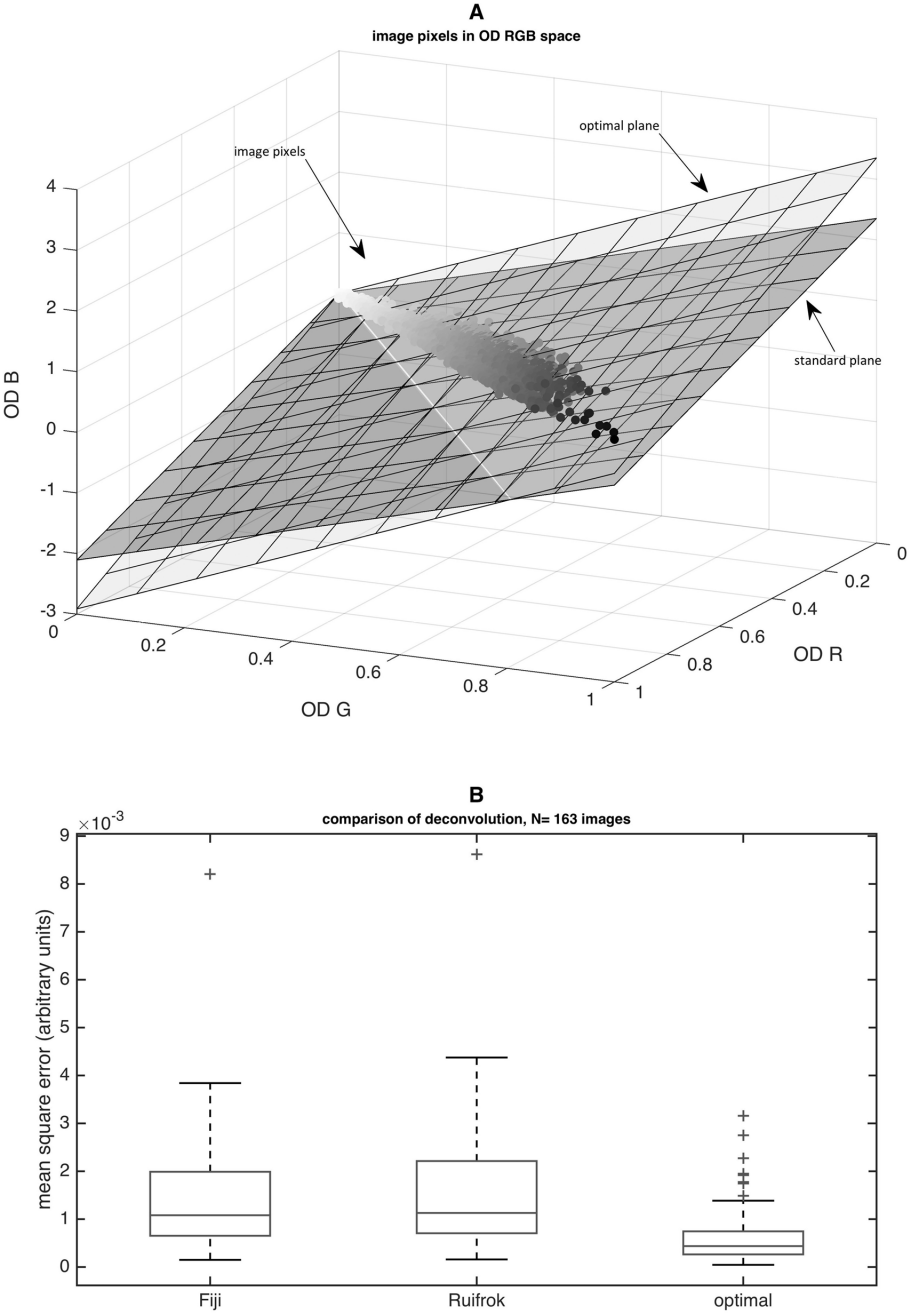
An optimized approach for color deconvolution based on principal component analysis minimizes the deconvolution residual. A) the image pixels of a given blue—brown immunostained image are plotted in the space of optical density color channels (OD R = red, OD G = green, OD B = blue). The commonly used standard color deconvolution vectors define a plane that roughly, but not optimally, approximates the data set (darker plane). The brighter plane shows the optimal plane containing the first and the second principal component vector of the actual image pixel dataset. B) Quantification of mean square error of the residual channel after color deconvolution with different deconvolution vectors. Boxes = 25th to 75th percentile, line = median, whiskers = most extreme data points except outliers, ‘+’ = outliers.

*M*_*Rui*_ is widely used for two-channel IHC images. However, image colors in histology depend on the dye chemical, sample preprocessing, staining protocol and scanning device. Considering this, it seems unlikely that *M*_*Rui*_ is the best choice for all H–DAB images. Consequently, we hypothesized that the result of color deconvolution can be improved by using the values in *M*_*Rui*_ and adjusting them to the image of interest. The standard deconvolution vectors ${\vec{H}}_{Rui}$ and ${\vec{D}}_{Rui}$ are part of a plane *P*_*Rui*_ that is shown as the ‘standard plane’ in Fig 1A. This plane does not optimally capture image pixel variability of a sample image, because it does not necessarily contain the first two principle component vectors of the image pixel data. By using principal component analysis (PCA), we constructed a plane that optimally approximates image pixel variability (‘optimal plane’ in Fig 1A). In Fig 1A *P*_*opt*_ is different from *P*_*Rui*_. *P*_*opt*_ is spanned by the two first principal component vectors of the image pixel color data (${\vec{C}}_{1}$ and ${\vec{C}}_{2}$). For an image composed of two colors (Hematoxylin (*H*) and DAB (*D*)), it follows that optimal color deconvolution vectors of these two colors ${\vec{H}}_{opt}$ and ${\vec{D}}_{opt}$ should be part of *P*_*opt*_ to minimize deconvolution error, i.e.

$$\begin{array}{c} {\vec{H}}_{opt}\in P_{opt} \end{array}$$ (1)

$$\begin{array}{c} {\vec{D}}_{opt}\in P_{opt} \end{array}$$ (2)

The third basis vector defines the residual information that cannot be expressed as linear combinations of the two stain vectors. Therefore, we define

$$\begin{array}{c} {\vec{\epsilon }}_{opt}⊥P_{opt} \end{array}$$ (3)

Consequently, an optimal color deconvolution matrix *M*_*opt*_ for an image composed of two colors is composed of the row vectors ${\vec{H}}_{opt},{\vec{D}}_{opt},{\vec{\epsilon }}_{opt}$.

On the plane *P*_*opt*_, the orientation of ${\vec{H}}_{opt}$ and ${\vec{D}}_{opt}$ is constrained by the color they represent: ${\vec{H}}_{opt}$ represents a bluish hue and ${\vec{D}}_{opt}$ a brown hue. The actual hues for a given image depend on many different parameters including chemical composition of the stain, incubation times of the stains and modalities of the scanning procedure. Because these parameters cannot be estimated from a given image, we use the following approach: For the data in a given image, we take the standard Hematoxylin (blue) and DAB (brown) vectors used by Ruifrok et al. (${\vec{H}}_{Rui},{\vec{D}}_{Rui}$) and define ${\vec{H}}_{opt}$ and ${\vec{D}}_{opt}$ as the projection of these vectors on the plane *P*_*opt*_. Thus, we ensure that the residual ${\vec{\epsilon }}_{opt}$ is minimal while the hue represented by the stain vectors stays as close as possible to the commonly used standard values.

Other groups have presented different optimization approaches for color deconvolution [22–25]. However, these methods rely on assumptions that are not applicable to IHC images and/or do not consider the commonly used—and well validated—standard values and no validation in large data sets is available for these alternative approaches.

### Measuring bivariate color maps in IHC images

**Fig 2:**
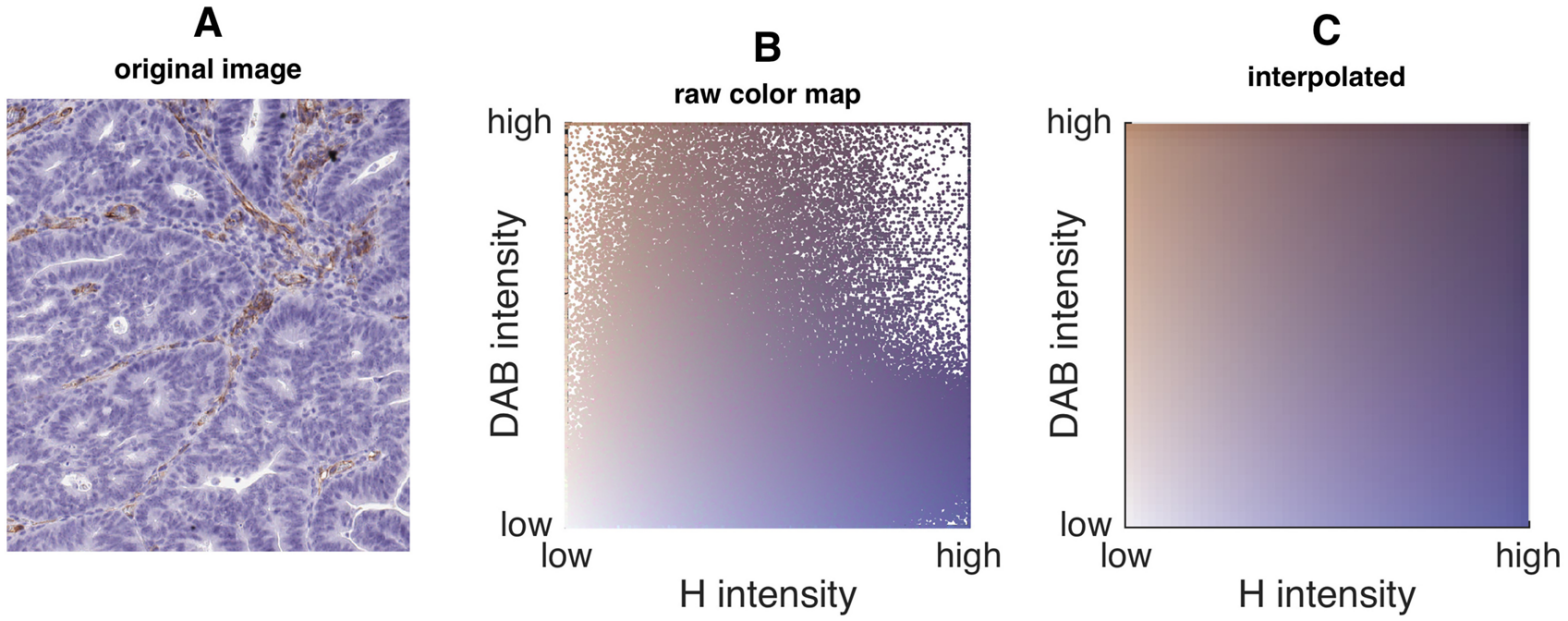
Measuring the bivariate color map in a given image. A) Original image detail (CD31 staining of tumor tissue), B) image pixels plotted in the space of the basis vectors of the two stain colors (H—DAB intensity space). C) Original bivariate color map interpolated from B. This color map contains all colors that are part of the original image. They are arranged in the H—DAB intensity space.

After color deconvolution, we plotted all image pixels in the space defined by the stain contributions. This is shown in Fig 2B. As can be seen, all image pixels are part of a fixed two-dimensional set of colors, i.e. a bivariate color map. Only small parts of this set of colors are not defined but they can be reconstructed by interpolation (Fig 2C).

### Contrast metric defined in the CIELAB space

To quantify perceptual difference (i.e. contrast) between two colors, we made use of the CIELAB color space (CIE International Commission on Illumination 1976 L*a*b*) [26, 27]. Images are usually digitally stored in the RGB color space but can be transformed to the CIELAB space by a non-linear transfer function *T*

$$\begin{array}{c} \left[L^{*},a^{*},b^{*}\right]=T\left(\left[R,G,B\right]\right) \end{array}$$ (4)

for red, green, blue color values [*R*, *G*, *B*]. The MATLAB documentation provides a definition of *T*. The CIELAB color space is perceptually uniform, which means that euclidean distances between colors in this space correspond to the color difference an average human observer perceives. The CIELAB color space is an established concept in science of human perception and is based on experimental data from human subjects [26, 27]. Its nonlinearity reflects the nonlinearity of the human visual system. The metric for color difference Δ*C* (i.e. perceived contrast) between an object of color $C_{1}=\left[L_{1}^{*},a_{1}^{*},b_{1}^{*}\right]$ and a reference color $C_{2}=\left[L_{2}^{*},a_{2}^{*},b_{2}^{*}\right]$ is defined as follows

$$\begin{array}{c} C=\sqrt{{\left(\Delta L^{*}\right)}^{2}+{\left(\Delta a^{*}\right)}^{2}+{\left(\Delta b^{*}\right)}^{2}} \end{array}$$ (5)

for

$$\begin{array}{ccc} \Delta L^{*} & = & ∥L_{1}^{*}-L_{2}^{*}∥\\ \Delta a^{*} & = & ∥a_{1}^{*}-a_{2}^{*}∥\\ \Delta b^{*} & = & ∥b_{1}^{*}-b_{2}^{*}∥ \end{array}$$

When comparing the ratio *R* of object-to-background contrast in two images, we calculated the contrast *C*_1_ for image 1 and *C*_2_ for image 2 and

$$\begin{array}{c} R=C_{1}/C_{2} \end{array}$$ (6)

Thus, if *C*_1_ > *C*_2_, then *R* represents the fold increase of object-to-background contrast. In the figures in the present paper, ‘L’, ‘a’, ‘b’ refer to the color space dimensions L*, a*, b*.

### Design and validation of perceptually improved color maps

To design a perceptually optimal bivariate color map, we extended established concepts on the optimal design of univariate color maps [28–31].

**Fig 3:**
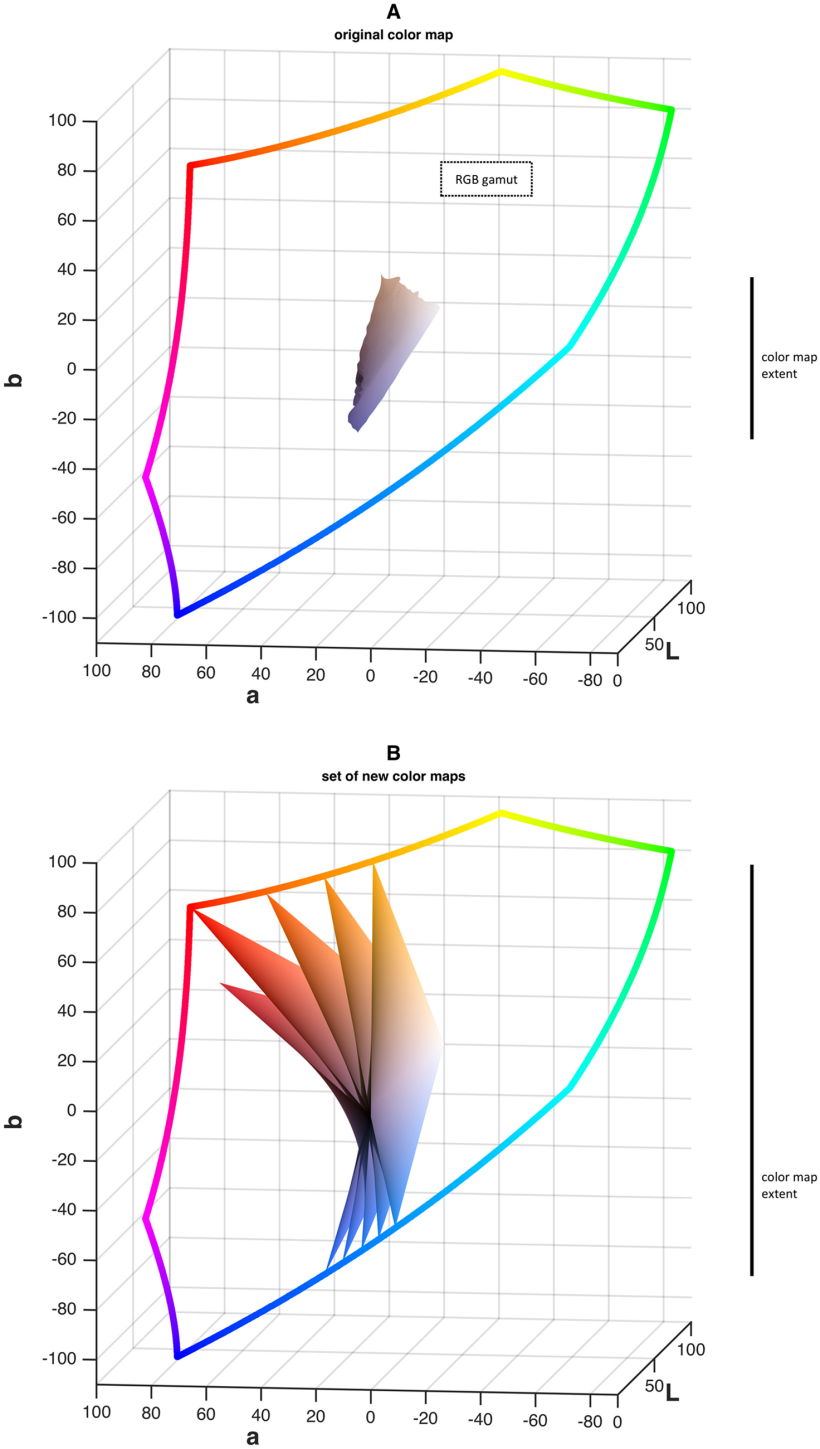
New color maps contain more colors than the original color map. In this figure, bivariate color maps are compared to the full sRGB gamut in CIELAB color space. A) Original color map extracted from the sample image. B) Set of 5 optimized color maps in the same view (color maps from Fig 4A–4E). It can be seen that the improved color maps occupy a larger part of the perceivable color space, maximizing visually transmittable information.

First, we examined the standard blue—brown color map in CIELAB space as shown in Fig 3A. It can be seen that compared to the full RGB gamut, this color map is small, i.e. contains only a small subset of colors. We strived to create an improved color map. We created alternative color maps as contiguous 2D planes in the 3D CIELAB space (Fig 3B). Our aim was to create a color map that was larger than the original color map, i.e. contained more visually distinct colors. Apart from the color map size, we defined the following criteria for an optimized color map:

1. A color map was defined by four corner points and a linearly interpolated plane in between. This restricted the shape of the color map and ensured that the resulting color map was perceptually linear.
2. Two diagonally opposing corners of the color map were set to black (#000000) and white (#FFFFFF), the two remaining corners were varied.
3. The color map should not contain more than two basic hues. This effectively limited the curvature of the color map because a highly curved plane tended to cross several distinct hues in CIELAB space (some of these highly curved color maps are included in S1 Fig).
4. Green and red hues should not be present in the same color map because of the high prevalence of red–green color vision deficiency (deuteranomaly).

We followed these rules and, in an iterative process, found that the criteria were only fulfilled by color maps containing a red—orange and a blue color. The result of this procedure can be seen in Fig 3B: the new color maps occupy a much larger proportion of the RGB gamut.

### Phantom images

**Fig 4:**
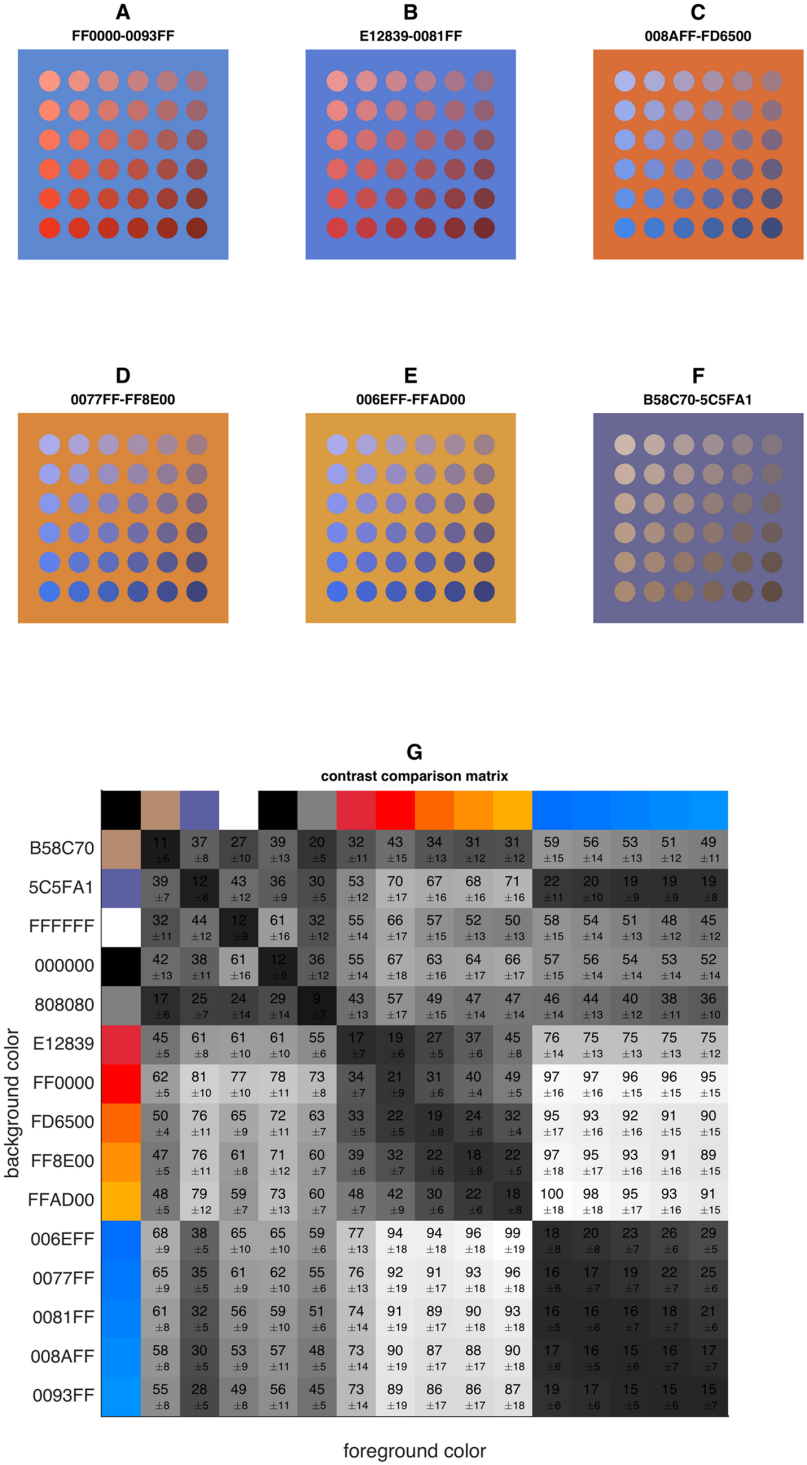
Foreground-background contrast in phantom images can be markedly increased by applying a new color map. A-F) A phantom image was colored in one of six bivariate color maps, the panel title indicate the hexadecimal codes of the foreground and background color (F represents a typical blue—brown standard color map while A-E represent new color combinations). G) 225 combinations of 15 distinct colors were pairwisely compared. The grayscale intensity and overlayed number indicate the perceptual contrast of phantom images that were digitally stained with the respective color map (numbers: mean ± standard deviation). All measurements were normalized to the maximum contrast. Reading example: The standard brown (#B58C70) on blue (#5C5FA1) color map resulted in a perceptual contrast that was 39% ± 7% of the maximally achieved contrast.

Next, we measured the achievable contrast with all possible combination of the five red/orange–blue color maps. Also, we included the standard blue and brown colors commonly used in H—DAB images and black, gray and white as controls. We created a phantom image of 6 ∗ 6 circles of different colors on a homogeneous background and applied the bivariate color maps to this image (Fig 4A–4E). We then evaluated perceptual contrast for each of these bivariate color maps in a phantom image (Fig 4G).

### Image source

For initial development of the method, we used a Hematoxylin (H)—3,3’-Diaminobenzidine (DAB) IHC image of CD31-positive blood vessels in a detail of an anonymized tumor image from our recent publication [20]. This image is included in the data we provide along with this paper. For validation of the method, we used a large dataset of publicly accessible, H—DAB IHC images from ProteinAtlas [32, 33]. To validate the proposed method, we chose eight commonly used cancer entities and commonly used IHC biomarkers: alpha-fetoprotein (AFP) in liver cancer, B-Raf proto-oncogene (BRAF) in melanoma, erb-b2 receptor tyrosine kinase 2 (ERBB2, HER2/neu) in breast cancer, estrogen receptor (ESR1) and progesterone receptor (PGR) in breast cancer, antigen KI-67 (MKI67, Ki67) in colorectal cancer and urothelial cancer and S100 calcium binding protein A1 (S100A1) in ovarial cancer. We then automatically retrieved all corresponding images URLs by a script via regular expressions and downloaded the images using a MATLAB script. In the code files associated with this study, we provide image URLs that were used. In total, we retrieved and analyzed N = 596 images without further preselection. In order to cut the margin around the actual tissue, the images were cropped before analysis. The license of the images allows use for non-profit research as long as the original citations are provided [32, 33].

For automatic measurement of foreground-to-background contrast in these images, we applied color deconvolution and thresholded the resulting DAB channel using Otsu’s automatic thresholding method [34]. Representative results of this procedure are shown in S2 Fig.

Lastly, we demonstrated that our proposed method can also be used to re-stain H&E or Giemsa stained images. For this purpose, we used fully anonymized images from our institution’s pathology archive (see ethics statement above).

### Color representation and figure preparation

Colors throughout this manuscript are represented by their hexadecimal codes, starting with “#”. For simulation of deuteranopia and tritanopia, we used the built-in plugin in Fiji / ImageJ [35]. All images in this paper were printed without any further modification of contrast, brightness, gamma value or colors.

### Computational implementation and licensing

We implemented all novel methods described in this paper in MATLAB (Mathworks, Natick, MA, USA). All source codes, image meta information and the sample image are publicly available on GitHub (DOI: 10.5281/zenodo.35083) under the MIT license (http://opensource.org/licenses/MIT). This repository contains an easily usable source code and example images and raw data used in our study. All other images are publicly available on ProteinAtlas (http://www.proteinatlas.org). All statistical analyses were performed using MATLAB and we used student’s t-test for evaluation of statistical significance.

Furthermore, we created a prototype of a digital pathology environment to view digitally re-stained sample images. For this implementation, we used VIPS [36] and OpenSeadragon 2.0.0 (http://openseadragon.github.io). This platform can be accessed on our project website (https://jnkather.github.com/HistoColor2D, source code DOI: 10.5281/zenodo.35091).

More information on the computational procedures is available in List A in S1 File. Raw data are available in S2 File.

## Results

### The proposed optimized stain unmixing method minimizes deconvolution error

The first step of the method we developed for this study was to optimally unmix stain channels of blue—brown IHC images by a novel approach (Fig 1A). We validated this method in N = 596 histological images and found that the quality of stain unmixing significantly increased, as evident by a decrease in mean square error (MSE) of the residual channel. As shown in Fig 1B, MSE for the ‘Fiji’ standard values was 0.0022, MSE for Ruifrok’s standard values was 0.0023 and MSE for the optimized method was 0.0007 (arbitrary units, t-test p-value <0.001 for optimal vs. either of both standard values).

### The standard color map in blue—brown images are poorly suited for human visual perception

To find an optimal color map, we created 225 color maps and measured perceptual contrast in a phantom image as shown in Fig 4A and 4B. We found that among all combinations of different hues, the standard brown (#B58C70) on blue (#5C5FA1) color map offered low perceptual contrast (only 39% ± 7% perceptual contrast of the best color map). The best hue combinations in terms of perceptual contrast was blue (#FFAD00) on orange (#006EFF) (set as 100% [±18%], see Fig 4G). Consequently, the new color map improved perceptual contrast by a factor of 2.56 (1/0.39). To visualize in which way the new hue combinations were superior to the standard hue combinations, we analyzed these maps in the CIELAB color space. In Fig 3, a blue—brown color map of a sample image (Fig 3A) is compared to five improved color maps (Fig 3B). It can be seen that the surface of the new color maps is larger than the surface of the original map, i.e. occupies more space in the CIELAB color space. This means that the new color maps can be used to encode more perceptionally distinct colors than the original one.

### Images can be digitally re-stained with arbitrary color maps

**Fig 5:**
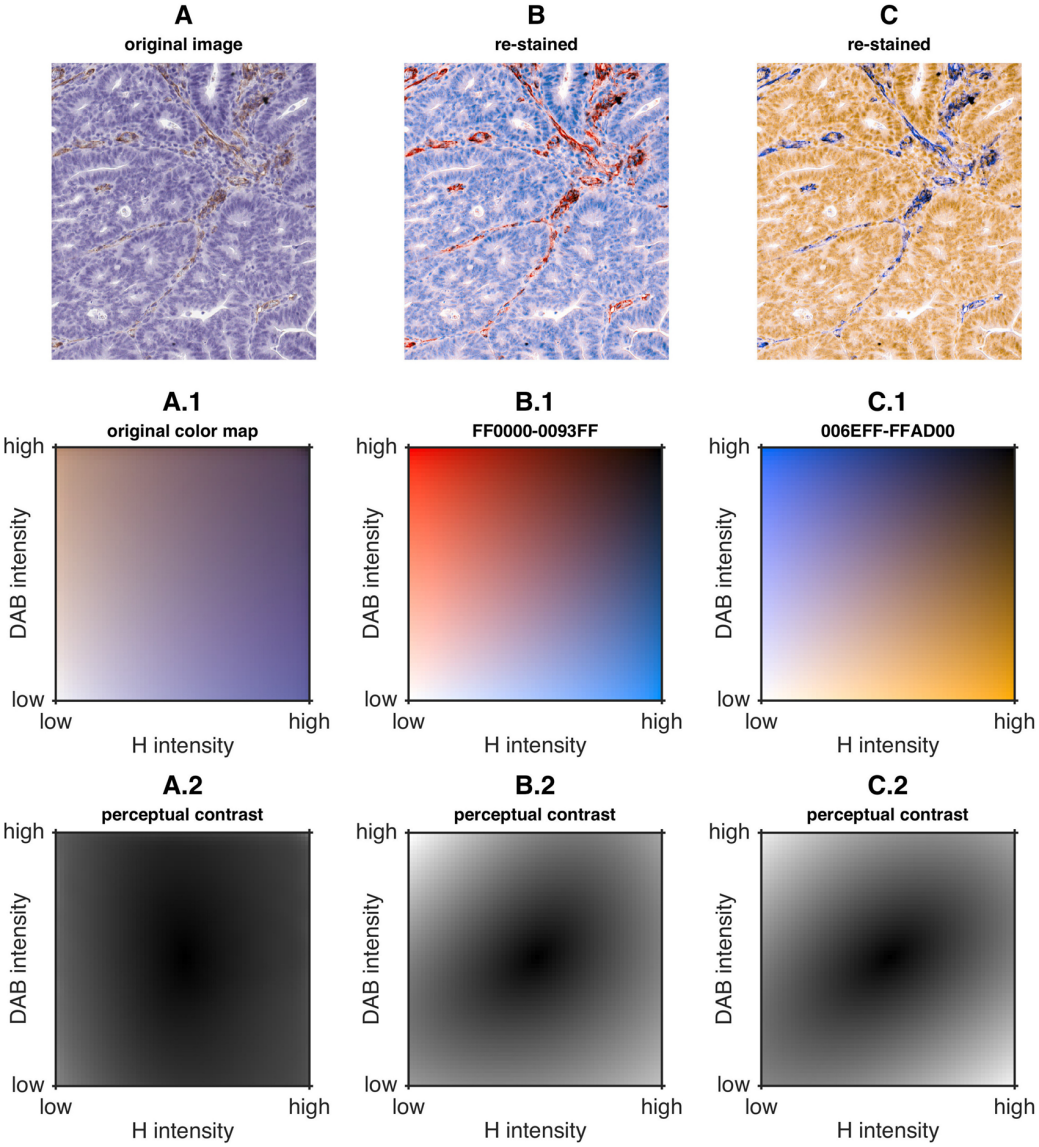
New color maps offer higher perceptual contrast than original color map in all relevant color map regions. A) Original image. B) Image re-stained with the optimized red (#FF0000)—blue (#0093FF) color map. C) Image re-stained with the optimized blue (#006EFF)—orange (#FFAD00) color map. A.1, B.1, C.1) Corresponding bivariate color maps. A.2, B.2, C.2) Intensity represents perceptual contrast (CIELAB distance) of A.1, B.2, C.1 relative to the center point. It can be seen that perceptual contrast of the original color map is low in almost all regions while the perceptual contrast of the new color maps is much higher.

After determining the optimal combination of colors for bivariate color maps, we applied these color maps to a sample image of a blood vessel in human colorectal cancer (Fig 5). In Fig 5A, we show a sample histological image which is re-stained in 5B and 5C. The corresponding color maps are shown in Fig 5A.1–C.1.

Furthermore, we quantified the perceptual contrast in each color map. This contrast can be measured as the perceptual distance of each color contained in the color map to a neutral element. We set the center point of the color map as neutral and computed perceptual contrast as described in the ‘Materials and Methods’ section. For example, for the blue (#006EFF) on orange (#FFAD00) color map, the result can be seen Fig 5C.2 as a gray scale coded plot. For the original brown (#B58C70) on blue (#5C5FA1) color map, the contrast of most colors was low (CIELAB maximum distance: 51.7, mean 22.9 ± 10.1). For the new color map blue (#006EFF) on orange (#FFAD00), the contrast was much higher in almost all regions, especially in the high-intensity regions of one of the two colors (CIELAB maximum distance: 82.6, mean 37.4 ± 16). For the other color combinations from Fig 4A–4E, maximum contrast and mean contrast are also higher than for the original blue—brown color map (see Table A in S1 File).

**Fig 6:**
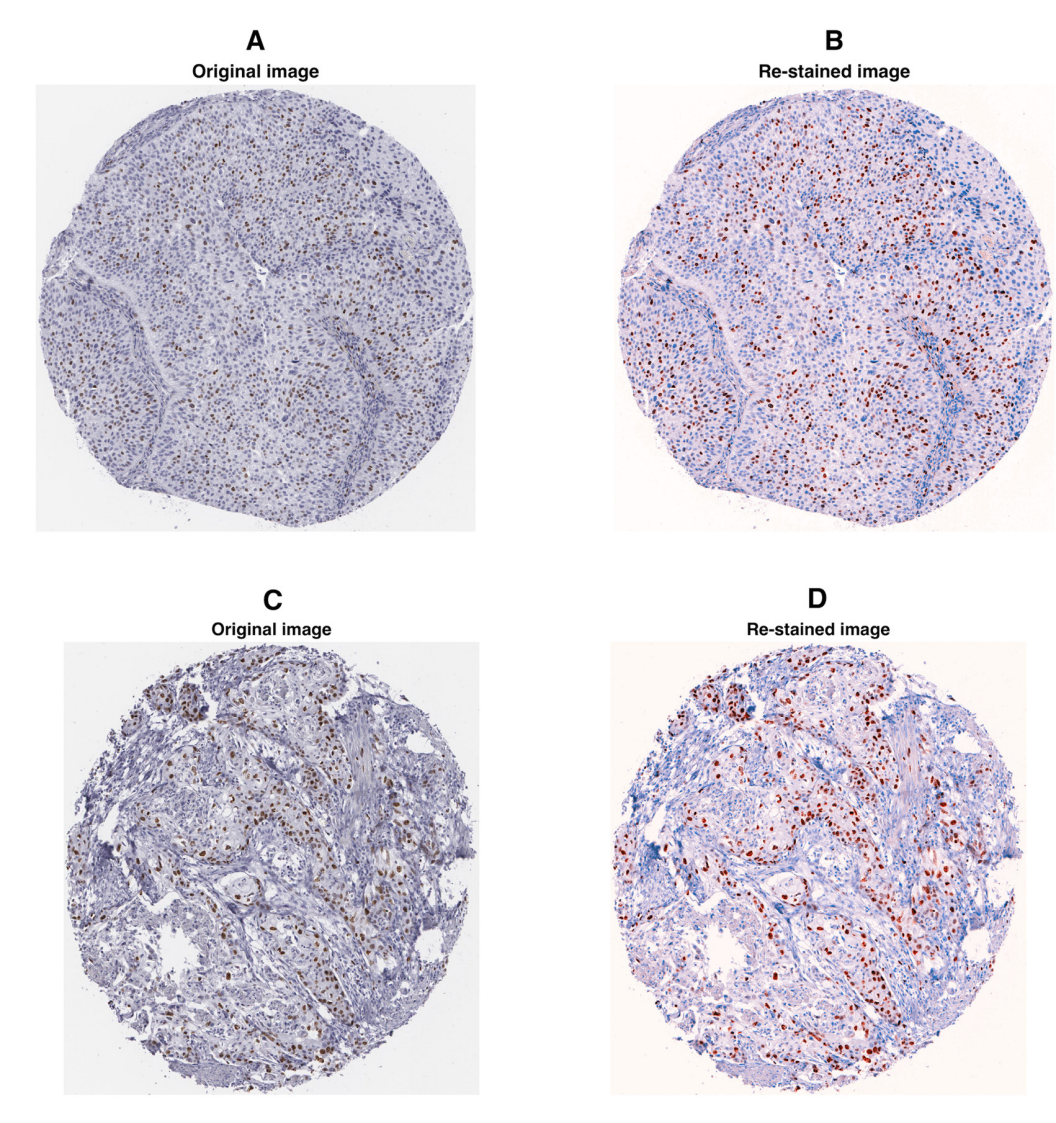
Digitally re-stained images of Ki67 stained samples. Two representative images from set ‘MKI67-uro’ (Ki67 in urothelial cancer). (A, C): original images, (B, D): re-stained images in red (#FF0000)—blue (#0093FF). It can be seen that the contrast of foreground (i.e. Ki67 positive cells) to background is improved after re-staining.

Finally, we used the described method to re-stain 8 sets of histological images of different human solid tumors stained for clinically relevant markers. In Fig 6, two examples from the set ‘MKI67-uro’ (Ki67 in urothelial cancer) are shown.

### Digital re-staining increases perceptual contrast in real histological images

**Fig 7:**
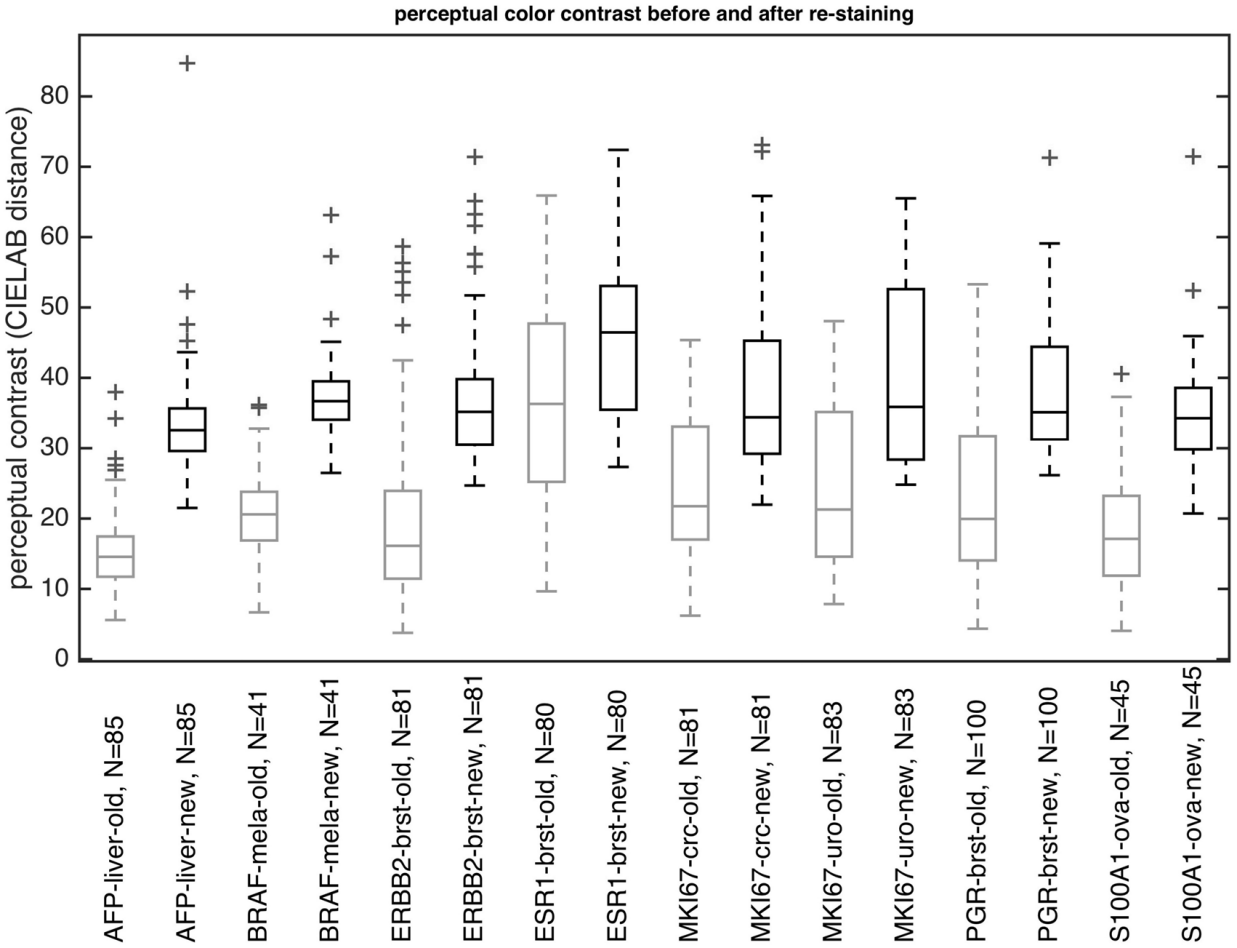
Digital re-staining markedly increases perceptual contrast in N = 596 actual histological images. Perceptual contrast before (gray boxes) and after (black boxes) color map improvement was measured in eight sets of histological images of human solid tumors (see ‘Materials and Methods’, total N = 596 samples). For all datasets, a pronounced increase of perceptual contrast can be seen. Boxes = 25th to 75th percentile, line = median, whiskers = most extreme data points except outliers, ‘+’ = outliers. The color map used for re-staining was blue (#006EFF)—orange (#FFAD00).

To clarify how digital re-staining of N = 596 actual histological images affects perceptual contrast, we measured object-to-background contrast in each of these images before and after the procedure. A representative result of this procedure is shown in S2 Fig. We found that contrast was increased in all datasets (Fig 7). Perceptual contrast of the new images was increased by 116.99% (AFP-liver), 78.7% (BRAF-mela), 82.22% (ERBB2-brst), 30.66% (ESR1-brst), 60.69% (MKI67-crc), 63.3% (MKI67-uro), 62% (PGR-brst) and 92.71% (S100A1-ova). The increases were highly significant (all t-test p-values <0.001, see Table B in S1 File for detailed measurements).

### Digital re-staining increases perceptual contrast for observers with impaired color vision

In a last step, we created simulations of the color map appearance to dichromatic observers. Dichromacy is the complete lack of one type of retinal cones, i.e. red, green or blue. Most persons with impaired color vision do not have a complete lack of one cone type, but rather a partial impairment. However, it is difficult to simulate partial color-vision deficiencies due to the wide variety, therefore we decided to use a well-established simulation of the most extreme case of complete dichromacy. We found that some of the proposed color maps were by far superior to the original blue—brown color map in this setting (e.g. see S3 Fig). Especially the most contrasted blue (#006EFF) on orange (#FFAD00) color map preserved perceptual contrast after dichromacy simulation.

### Generalization of the method and application to other types of staining

**Fig 8:**
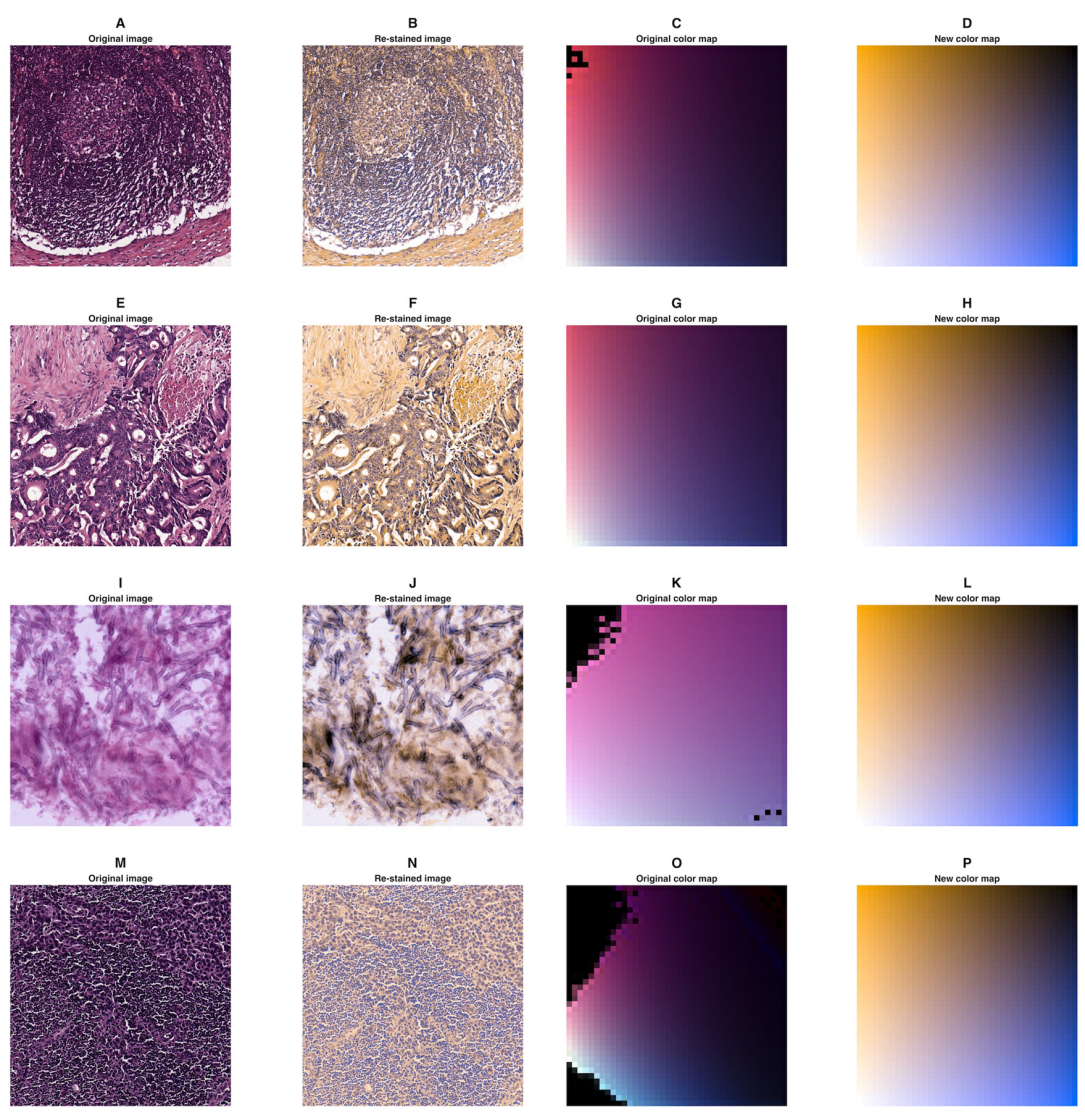
Examples of digitally re-stained H&E images. (A-D) Normal lymph node tissue, (E-H) colorectal carcinoma tissue, (I-L) aspergillus, (M-P) breast cancer tissue surrounded by lymph node tissue. For each sample, the original image, the re-stained image and the original and resulting color map are shown. The color map used for re-staining was orange (#FFAD00)—blue (#006EFF). Sizes are: (A) 742 ∗ 742*μ*m, (E) 742 ∗ 742*μ*m, (N) 594 ∗ 594*μ*m. (I) had no specified size (image source see List B in S1 File).

**Fig 9:**
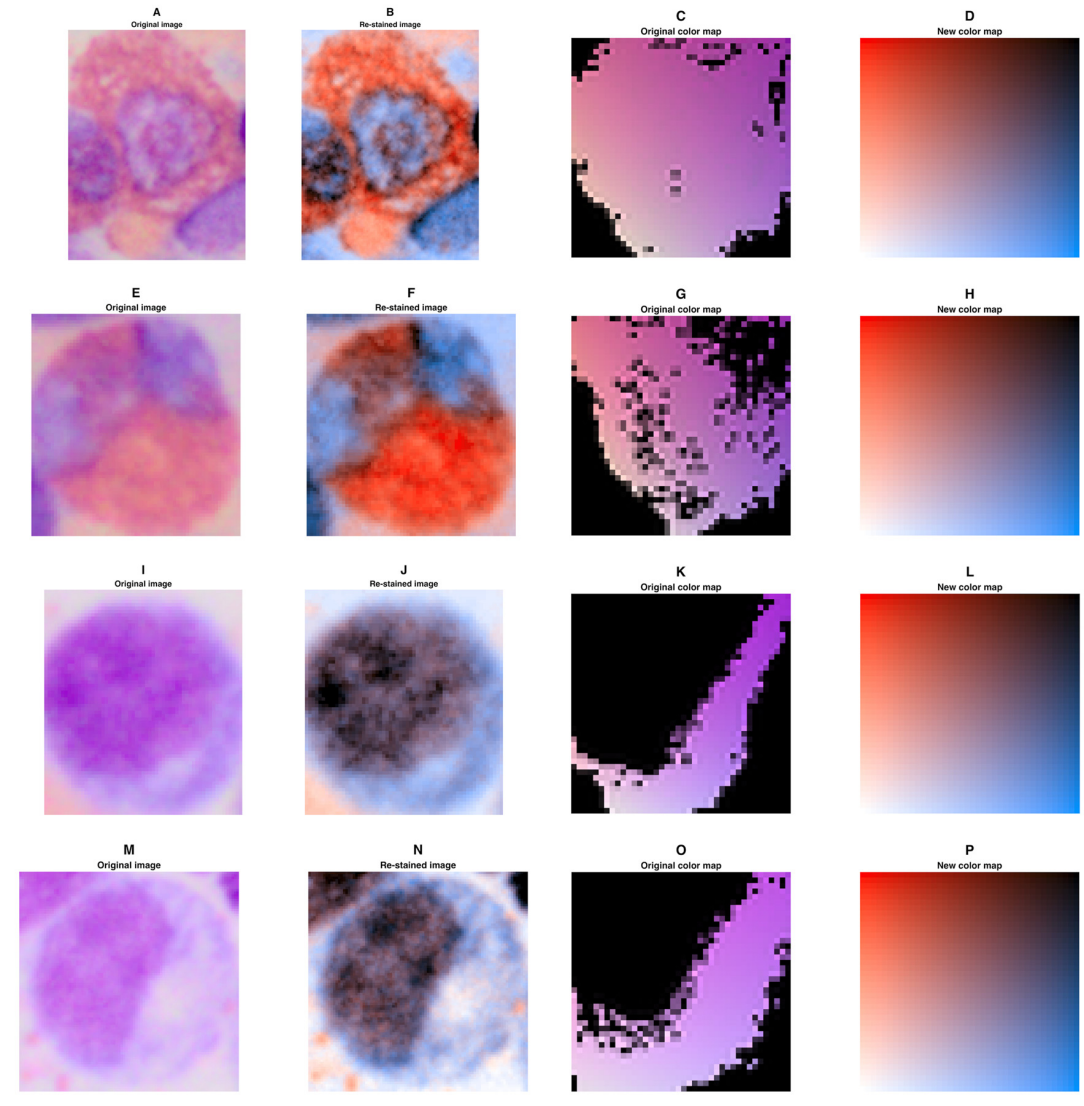
Examples of digitally re-stained Giemsa stained images. (A, E, I, M) Single cells that were extracted from a Giemsa-stained bone marrow biopsy image. (B, F, J, N) Digitally re-stained image using red (#FF0000)—blue (#0093FF). (C, G, K, O) Original color maps. It can be seen that these color maps are not continuous. (D, H, L, P) Discontinuities are inherently corrected by the re-staining procedure and perceptual contrast is improved in the new color maps.

The focus of our work was to enhance perceptual foreground-to-background contrast in H-DAB IHC images. However, the method we present can also be applied to other types of staining. As a proof of principle, we have arbitrarily chosen four sample H&E images and re-stained them using a orange—blue color map (Fig 8). Unlike in H-DAB IHC images, foreground and background are not clearly defined in these H&E images. Yet, it can be appreciated that some structures in the image are more clearly visible after re-staining (e.g. aspergillus in Fig 8I and 8J) and that virtual re-staining corrects discontinuities in the original color maps (e.g. Fig 8C, 8K and 8O). Also, virtual re-staining inherently corrects for illumination: While the original images in Fig 8A, 8E, 8I and 8M are not equally bright, these differences are removed after virtual re-staining (Fig 8B, 8F, 8J and 8N). Furthermore, we found that virtual re-staining can be successfully applied to Giemsa-stained images of bone marrow biopsies. In Fig 9, it can be seen that contrast between cytoplasm and cell nucleus is markedly increased by re-staining Giemsa stained images of human bone marrow.

## Discussion

### Digital re-staining improves perceptual contrast in IHC images

By using the method we describe in this study, pathologists can be enabled to visually perceive more information about the microscopic structures in an image. The main reason is that the bivariate color maps presented in this study are optimized for human visual perception so that a human observer is enabled to extract more information from looking at a processed image. Using our method, the object-to-background contrast can be markedly increased by a factor of 2.56 in phantom images and up to a factor of 2.17 (equals an improvement of approx. 117%) in eight sets of real images.

It is important to note that this method does not alter objective information content of images: The information content of a histological image is determined by sample processing, staining methods and optical properties of the digitization system. However, as shown above, the way of displaying histological images to human observers is not well suited for transmitting a maximal amount of the available information. The reason for this shortcoming is that of all colors distinguishable by the human visual system, histological images use only a very small subset. Using the method we present in this study, the numbers of distinct colors in a given image can be retrospectively optimized so that a higher percentage of the available contrast information can be perceived by an observer.

### Compliance with digital image ethics and comparison to other medical imaging methods

Scientific data in general, and digital images in particular, should not be arbitrarily ‘beautified’. Strict standards are necessary to ensure that images are not changed in any way that changes the underlying data. Cromey et al. have proposed a widely accepted set of rules for handling digital images [37]. One of the guidelines they give is that the use of software filters to improve image quality is usually not recommended. Yet, digital image enhancement according to objective protocols is widely used in medical imaging. Diagnostic imaging technologies such as CT and MR imaging produce data that is heavily edited by smoothing kernels and windowing to make most of the information visible to human observers. Furthermore, CT and MR imaging data are routinely digitally colored using a determined color map [13–15]. This has been shown to increase diagnostic accuracy in some settings [12].

Also, as explained above, immunostained histological images are in fact bivariate data sets where two stain intensities are displayed as a color that derives from a determined mixture of the two stain contributions. Normally, this bivariate color map cannot be explicitly viewed by an observer but is implicitly represented in the image. We employ a novel technique to extract the inherent bivariate color map from a given image. Having taken this abstraction step, the image can be treated as any two-dimensional dataset displayed with a bivariate color map. For this general case, there is a lot of experience in the literature [38, 39]. Several authors have shown how to optimally display bivariate color sets and there are a number of practical instructions on how to design bivariate color maps for scientific data [40, 41]. We employ this knowledge on datasets from histological images to re-create a digitally re-stained image.

Furthermore, we encourage users of our technique to keep the original data as pointed out by Cromey et al. [37]. If users are in doubt whether structures in the enhanced image are of diagnostic relevance, an enhanced and an original image could be viewed side by side to arrive at an accurate diagnostic decision.

In summary, we offer a strictly deterministic and reproducible way of automatically extracting the implicit color map and applying a new bivariate color map to a given data set, i.e. an image. Our method does not arbitrarily ‘beautify’ a given image but is a way of making a larger percentage of the existing information available to the visual system of a human observer. These procedures are already standard approaches in other medical imaging entities but have never been applied and validated in the context of histopathological imaging.

### Digital re-staining mitigates handicap of pathologists with impaired color vision

Approximately 8% of all males and 0.4% of all females are affected by anomalous color vision [42, 43]. Most of them suffer from red-green deficiency (deuteranomaly) that is X-chromosomally linked [42, 44]. To our knowledge, it is presently unclear whether diagnoses made by deuteranomalous pathologists differ from those made by fully trichromatic pathologists [45]. However, it is conceivable that deuteranomalous observers might evaluate a colored image differently than the trichromatic population. We show that while some color maps are preserved after dichromacy simulation, other color maps lose most of their perceptual contrast. This is especially true for the standard blue—brown color maps used in immunohistology (S3 Fig, panel A). Other color maps, such as the proposed blue—yellow or red—blue maps are more robust with respect to color blindness simulation (S3 Fig, panels B and C). Thus, digital re-staining of histological images offers the possibility to apply color maps that mitigate the handicap of pathologists with impaired color vision by using color coding that can be separated by the individual cone dysfunction. One color in the blue area of the color space for the background—as in H-DAB-histological images—and the other color in the orange or red area improves discrimination of stained cells and assists observers with color deficiency in their image perception.

### Limitations and perspectives

In the present study, we developed and evaluated a method to digitally re-stain histological images. We showed that our improved color maps markedly increase perceptual contrast in clinically relevant images. However, our approach does not guarantee a global optimum for a new color map. In further studies, it would be worthwhile to combine our approach with a sophisticated multi-parameter optimizer (such as the one described by Hutter et al., see [46]). Also, as a perspective, it would be interesting to quantify the diagnostic accuracy for different color maps in different common tasks in histopathology in future studies. Apart from histology, it is conceivable to apply the described method to other imaging modalities, such as MR imaging or MR microscopy [47].

### Application in a typical diagnostic setting

We envision that the presented method can be incorporated in digital pathology image viewing systems. For example, in a typical histopathological diagnostic setting, a pathologist might want to view and count Ki67 positive cells in a Hematoxylin—DAB stained section of tumor tissue. For some cells, the decision whether or not they are Ki67 positive is usually easy. However, there might be some cells for which the decision is not clear because the pathologist cannot exactly visually discriminate the color hues. This scenario is possible even if the staining procedure and optical properties of the microscope are optimal and even if the pathologist has a perfectly normal color vision (trichromacy). In this case, one reason why the pathologist in this scenario cannot exactly discriminate the Ki67 status of a cell is that his or her visual system physiologically cannot discriminate the *colors* in the image.

In a digital pathology environment, the pathologist could then make use of alternative color maps and digitally re-stain the image in real time. As common in radiology, the source image and the modified image could be viewed side-by-side. In this situation, the pathologist could choose a color map that maximizes his or her ability to recognize objects in the image. Therefore, his or her diagnostic ability to discriminate Ki67 positive from Ki67 negative cells would be increased. In the end, this would potentially reduce ambiguities in visual evaluation of histopathological samples, increase diagnostic accuracy and in the end benefit the patient.

## Supporting Information

S1 Fig Alternative set of new color maps. The highly curved color maps have an even larger surface than the ones we actually used in our study. However, they were excluded according to the described criteria because their high curvature led to artifacts in the resulting images.
(TIFF)

S2 Fig Contrast improvement in an image from ‘MKI67-crc’ (Ki67 in colorectal cancer). A) Original image, B) Digitally re-stained image, C) segmented DAB intensity channel, D-E) Foreground-to-background contrast for the original colors and the colors of the re-stained images. F) The cleaned foreground was created by removing small objects from the raw foreground mask.
(TIFF)

S3 Fig Color blindness simulation. A) Original image, B) and C) show digitally re-stained images. Below, the three images are shown after deuteranopia simulation and after tritanopia simulation (same order as top row). It can be seen that contrast in B and C is largely preserved after simulation, while contrast in A is largely lost after simulation.
(TIFF)

S1 File Additional experimental data and parameters. **Table A**: Contrast metrics for original and new color maps. **Table B**: Perceptual contrast improvement of objects in histological images. **List A**: Details of the computational procedures. **List B**: Image credit.
(PDF)

S2 File Raw data. This ZIP file contains all raw data used for the experiments. Folder ‘data’ contains a list of colors used for the color maps, folder ‘image-URLs’ contains all image URLs for ProteinAtlas images and folder ‘sample_images’ contains image files for all remaining images.
(ZIP)

## References

[1] Werner M, Chott A, Fabiano A, Battifora H. Effect of formalin tissue fixation and processing on immunohistochemistry. Am J Surg Pathol. 2000;24(7):1016–1019. 10.1097/00000478-200007000-00014 10895825

[2] Walker RA. Quantification of immunohistochemistry—issues concerning methods, utility and semiquantitative assessment I. Histopathology. 2006;49(4):406–410. 10.1111/j.1365-2559.2006.02514.x 16978204

[3] Taylor CR, Levenson RM. Quantification of immunohistochemistry—issues concerning methods, utility and semiquantitative assessment II. Histopathology. 2006;49(4):411–424. 10.1111/j.1365-2559.2006.02513.x 16978205

[4] Rasmussen OF, Rudbeck L. Immunohistochemistry: A Dako Perspective. In: Handbook of Practical Immunohistochemistry. New York: Springer; 2015 p. 57–67.

[5] Ntziachristos V. Going deeper than microscopy: the optical imaging frontier in biology. Nat Methods. 2010;7(8):603–614. 10.1038/nmeth.1483 20676081

[6] Glatz-Krieger K, Glatz D, Mihatsch MJ. Virtual slides: high-quality demand, physical limitations, and affordability. Hum Pathol. 2003;34(10):968–974. 10.1053/S0046-8177(03)00348-4 14608529

[7] Bautista PA, Hashimoto N, Yagi Y. Color standardization in whole slide imaging using a color calibration slide. J Path Informatics. 2014;5:4 10.4103/2153-3539.126153 PMC3952402 24672739

[8] Crowley RS, Naus GJ, Stewart J, Friedman CP. Development of visual diagnostic expertise in pathology—an information-processing study. J Am Med Inform Assn. 2003;10(1):39–51. 10.1197/jamia.M1123 PMC150358 12509356

[9] Krupinski EA, Graham AR, Weinstein RS. Characterizing the development of visual search expertise in pathology residents viewing whole slide images. Hum Pathol. 2013;44(3):357–64. 10.1016/j.humpath.2012.05.024 22835956

[10] Judd DB, Wyszecki G. Color in Business, Science and Industry In: Wiley Series in Pure and Applied Optics. 3rd ed New York: Wiley-Interscience; 1975 p. 388.

[11] Kimpe T, Tuytschaever T. Increasing the number of gray shades in medical display systems–how much is enough? J Digit Imaging. 2007;20(4):422–32. 10.1007/s10278-006-1052-3 17195900 PMC3043920

[12] Saba L, Argiolas GM, Raz E, Sannia S, Suri JS, Siotto P, et al Carotid artery dissection on non-contrast CT: Does color improve the diagnostic confidence? Eur J Radiol. 2014;83(12):2288–2293. 10.1016/j.ejrad.2014.09.001 25306107

[13] Kumar V, Udayashankara V. Automatic Colour Transfer Function Generation and 3D Reconstruction of DICOM Images. In: Proceedings of the Second National Conference on Computational Control Systems and Optimization; 2013. p. 10–15.

[14] Silverstein JC, Parsad NM, Tsirline V. Automatic perceptual color map generation for realistic volume visualization. J Biomed Inform. 2008;41(6):927–35. 10.1016/j.jbi.2008.02.008 18430609 PMC2651027

[15] Horvat D, Žalik B, Rupnik MS, Mongus D. Visualising the Attributes of Biological Cells, Based on Human Perception In: Human-Computer Interaction and Knowledge Discovery in Complex, Unstructured, Big Data. Berlin/Heidelberg: Springer; 2013 p. 386–399.

[16] Khan AM, Rajpoot N, Treanor D, Magee D. A nonlinear mapping approach to stain normalization in digital histopathology images using image-specific color deconvolution. IEEE T Bio-Med Eng. 2014;61(6):1729–38. 10.1109/TBME.2014.2303294 24845283

[17] Bautista PA, Yagi Y. Staining correction in digital pathology by utilizing a dye amount table. J Digit Imaging. 2015;28(3):283–94. 10.1007/s10278-014-9766-0 25561073 PMC4441690

[18] Reyes-Aldasoro CC. Retrospective shading correction algorithm based on signal envelope estimation. Electron Lett. 2009;45(9):454 10.1049/el.2009.0320

[19] Landini G, Perryer G. Digital enhancement of haematoxylin- and eosin-stained histological images for red-green colour-blind observers. J Microsc. 2009;234(3):293–301. 10.1111/j.1365-2818.2009.03174.x 19493108

[20] Kather J, Marx A, Reyes-Aldasoro C, Schad L, Zöllner F, Weis CA. Continuous representation of tumor microvessel density and detection of angiogenic hotspots in histological whole-slide images. Oncotarget. 2015;6(22):19163–76. 10.18632/oncotarget.4383 26061817 PMC4662482

[21] Ruifrok AC, Johnston DA. Quantification of histochemical staining by color deconvolution. Anal Quant Cytol Histol. 2001;23(4):291–299. 11531144

[22] Xu J, Xiang L, Wang G, Ganesan S, Feldman M, Shih NN, et al Sparse Non-negative Matrix Factorization (SNMF) based color unmixing for breast histopathological image analysis. Comput Med Imag Grap. 2015; Available from: 10.1016/j.compmedimag.2015.04.002 10.1016/j.compmedimag.2015.04.002 25958195

[23] Rabinovich A, Agarwal S, Laris C, Price JH, Belongie SJ. Unsupervised Color Decomposition Of Histologically Stained Tissue Samples. In: Thrun S, Saul LK, Schölkopf B, editors. Advances in Neural Information Processing Systems 16. MIT Press; 2004 p. 667–674.

[24] Trahearn N, Snead D, Cree I, Rajpoot N. Multi-class stain separation using independent component analysis. Proc SPIE. 2015;9420:94200J–94200J–11.

[25] Liu MC, Robertson M. Method and apparatus for stain separation in digital pathology images. US Patent; 2014. Available from: https://www.google.com/patents/US8744165

[26] Jain AK. Fundamentals of Digital Image Processing. Upper Saddle River, NJ: Prentice Hall; 1989.

[27] Westland S, Ripamonti C, Cheung V. Computational colour science using MATLAB. Hoboken, NJ: John Wiley & Sons; 2012.

[28] Harrower M, Brewer CA. ColorBrewer. org: an online tool for selecting colour schemes for maps. Cartogr J. 2003;40(1):27–37. 10.1179/000870403235002042

[29] Niccoli M. Geophysical tutorial: How to evaluate and compare color maps. The Leading Edge. 2014;33(8):910–912. 10.1190/tle33080910.1

[30] Borland D, Taylor RM II. Rainbow color map (still) considered harmful. IEEE Comput Graph. 2007;27(2):14–17. 10.1109/MCG.2007.323435 17388198

[31] Moreland K. Diverging color maps for scientific visualization In: Advances in Visual Computing. Berlin/Heidelberg: Springer; 2009 p. 92–103.

[32] Pontén F, Jirström K, Uhlen M. The Human Protein Atlas–a tool for pathology. J Pathol. 2008;216(4):387–93. 10.1002/path.2440 18853439

[33] Uhlen M, Fagerberg L, Hallstrom BM, Lindskog C, Oksvold P, Mardinoglu A, et al Tissue-based map of the human proteome. Science. 2015;347(6220):1260419–1260419. 10.1126/science.1260419 25613900

[34] Otsu N. A threshold selection method from gray-level histograms. Automatica. 1975;11(285–296):23–27.

[35] Walter T, Shattuck DW, Baldock R, Bastin ME, Carpenter AE, Duce S, et al Visualization of image data from cells to organisms. Nat Methods. 2010;7(3):S26–41. 10.1038/nmeth.1431 20195255 PMC3650473

[36] Martinez K, Cupitt J. VIPS—a highly tuned image processing software architecture. IEEE ICIP. 2005;p. 574–577.

[37] Cromey DW. Avoiding twisted pixels: ethical guidelines for the appropriate use and manipulation of scientific digital images. Sci Eng Ethics. 2010;16(4):639–67. 10.1007/s11948-010-9201-y 20567932 PMC4114110

[38] Eyton JR. Complementary-color, two-variable maps. Ann Assoc Am Geogr. 1984;74(3):477–490. 10.1111/j.1467-8306.1984.tb01469.x

[39] Teuling AJ, Stöckli R, Seneviratne SI. Bivariate colour maps for visualizing climate data. Int J Climatol. 2011;31(9):1408–1412. 10.1002/joc.2153

[40] Brewer CA. Color use guidelines for mapping and visualization. vol. 2. London: Pergamon; 1994. p. 123–148.

[41] Stevens J. Bivariate Choropleth Maps: A How-to Guide; 2015. Available from: http://joshuastevens.net/cartography/make-a-bivariate-choropleth-map

[42] MacHado GM, Oliveira MM, Fernandes LA. A physiologically-based model for simulation of color vision deficiency. IEEE T Vis Comput Gr. 2009;15(6):1291–1298. 10.1109/TVCG.2009.113 19834201

[43] Cole BL. Assessment of inherited colour vision defects in clinical practice. Clin Exp Optom. 2007;90(3):157–175. 10.1111/j.1444-0938.2007.00135.x 17425762

[44] Birch J. Diagnosis of defective colour vision. Oxford: Oxford University Press; 1993.

[45] Spalding JAB. Confessions Of A Colour Blind Physician. Clin Exp Optom. 2004;87(4–5):344–349. 10.1111/j.1444-0938.2004.tb05065.x 15312038

[46] Hutter M, Steiger M, Bernard J, Zurloh C, Kohlhammer J. Interactive Multi-Criteria Optimization of 2D Color Maps; 2014. Available from: http://www.gris.informatik.tu-darmstadt.de/˜jubernar/posters/vmv2014_mh_abstract.pdf

[47] Gottwald E, Kleintschek T, Giselbrecht S, Truckenmuller R, Altmann B, Worgull M, et al Characterization of a chip-based bioreactor for three-dimensional cell cultivation via Magnetic Resonance Imaging. Z Med Phys. 2013;23(2):102–110. 10.1016/j.zemedi.2013.01.003 23410914

