# Supplementary figures and images for: New Colors for Histology: Optimized Bivariate Color Maps Increase Perceptual Contrast in Histological Images
Source: PLoS One. 2015 Dec 30;10(12):e0145572. doi: 10.1371/journal.pone.0145572 (PMC4696851; doi:10.1371/journal.pone.0145572)

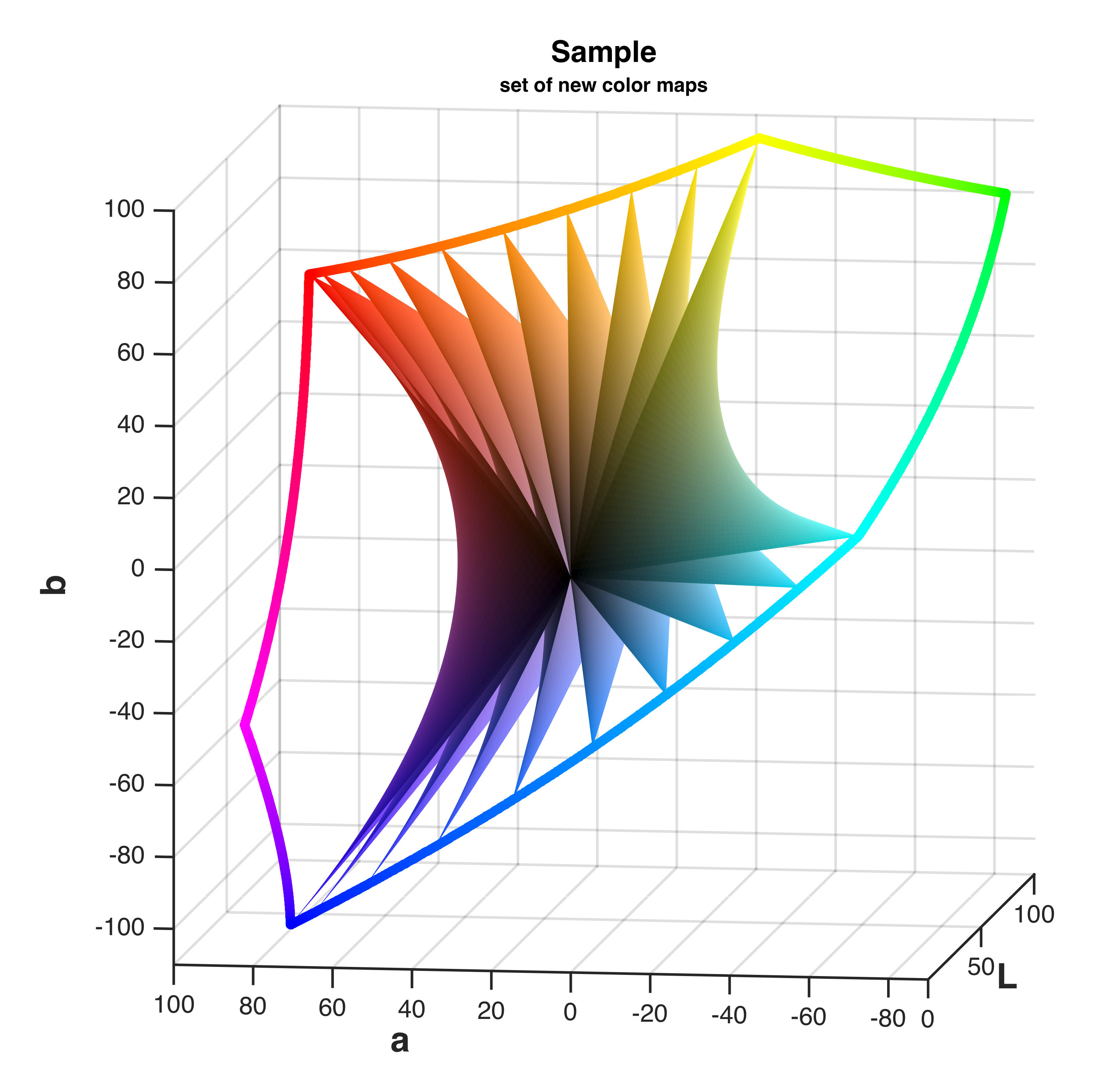

Supplement: S1 Fig — The highly curved color maps have an even larger surface than the ones we actually used in our study. However, they were excluded according to the described criteria because their high curvature led to artifacts in the resulting images. (TIFF) [file pone.0145572.s001.tiff]

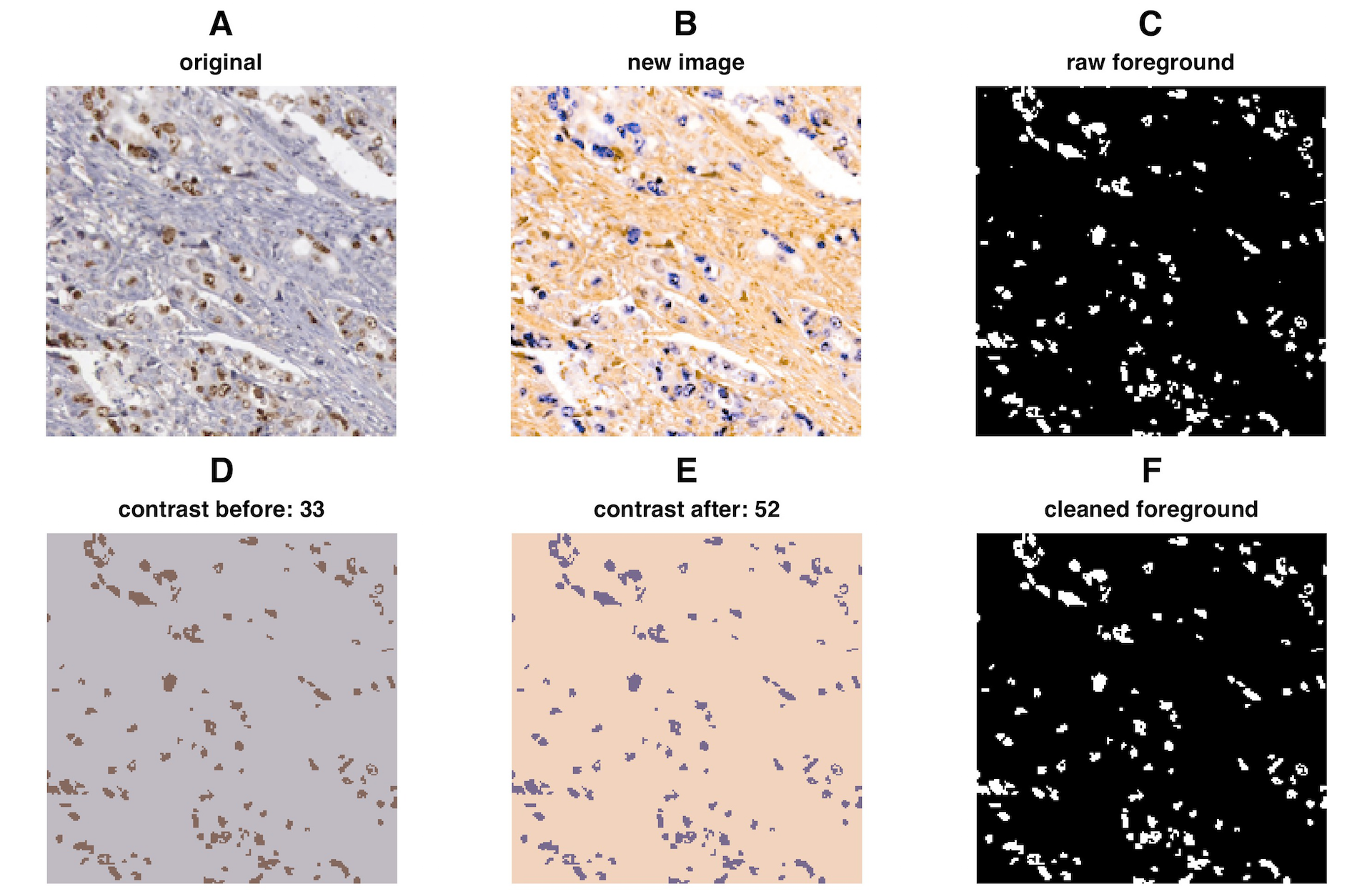

Supplement: S2 Fig — A) Original image, B) Digitally re-stained image, C) segmented DAB intensity channel, D-E) Foreground-to-background contrast for the original colors and the colors of the re-stained images. F) The cleaned foreground was created by removing small objects from the raw foreground mask. (TIFF) [file pone.0145572.s002.tiff]

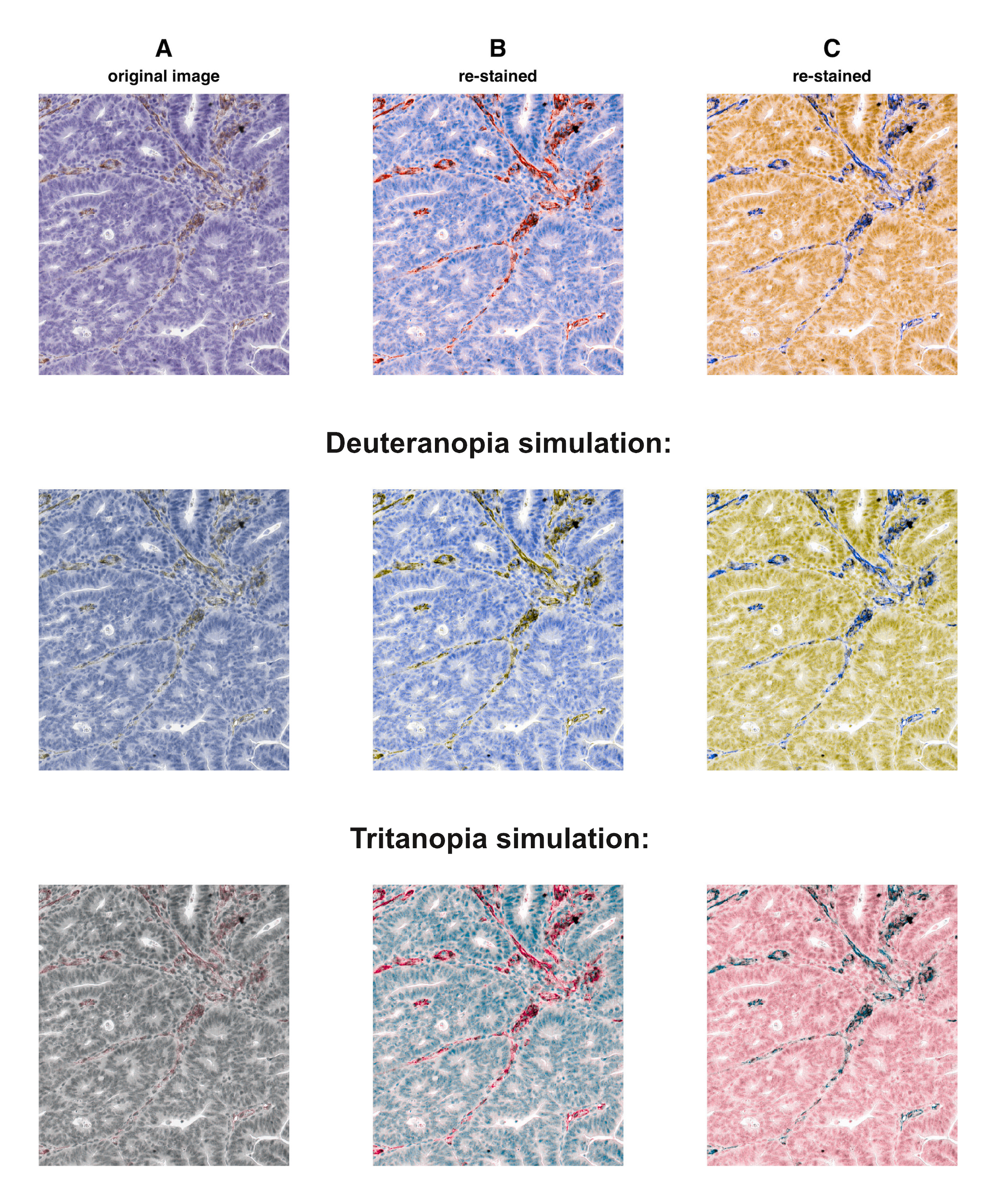

Supplement: S3 Fig — A) Original image, B) and C) show digitally re-stained images. Below, the three images are shown after deuteranopia simulation and after tritanopia simulation (same order as top row). It can be seen that contrast in B and C is largely preserved after simulation, while contrast in A is largely lost after simulation. (TIFF) [file pone.0145572.s003.tiff]

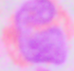

Supplement: S2 File — This ZIP file contains all raw data used for the experiments. Folder ‘data’ contains a list of colors used for the color maps, folder ‘image-URLs’ contains all image URLs for ProteinAtlas images and folder ‘sample_images’ contains image files for all remaining images. (ZIP) [file pone.0145572.s005.zip › S2_file/sample_images/Orig_Cell_Pos_X1013-Y558-W74-H71.png]

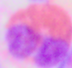

Supplement: S2 File — This ZIP file contains all raw data used for the experiments. Folder ‘data’ contains a list of colors used for the color maps, folder ‘image-URLs’ contains all image URLs for ProteinAtlas images and folder ‘sample_images’ contains image files for all remaining images. (ZIP) [file pone.0145572.s005.zip › S2_file/sample_images/Orig_Cell_Pos_X1083-Y379-W72-H68.png]

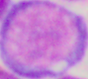

Supplement: S2 File — This ZIP file contains all raw data used for the experiments. Folder ‘data’ contains a list of colors used for the color maps, folder ‘image-URLs’ contains all image URLs for ProteinAtlas images and folder ‘sample_images’ contains image files for all remaining images. (ZIP) [file pone.0145572.s005.zip › S2_file/sample_images/Orig_Cell_Pos_X1173-Y1780-W88-H79.png]

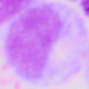

Supplement: S2 File — This ZIP file contains all raw data used for the experiments. Folder ‘data’ contains a list of colors used for the color maps, folder ‘image-URLs’ contains all image URLs for ProteinAtlas images and folder ‘sample_images’ contains image files for all remaining images. (ZIP) [file pone.0145572.s005.zip › S2_file/sample_images/Orig_Cell_Pos_X1237-Y418-W89-H89.png]

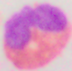

Supplement: S2 File — This ZIP file contains all raw data used for the experiments. Folder ‘data’ contains a list of colors used for the color maps, folder ‘image-URLs’ contains all image URLs for ProteinAtlas images and folder ‘sample_images’ contains image files for all remaining images. (ZIP) [file pone.0145572.s005.zip › S2_file/sample_images/Orig_Cell_Pos_X1242-Y1621-W72-H71.png]

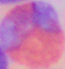

Supplement: S2 File — This ZIP file contains all raw data used for the experiments. Folder ‘data’ contains a list of colors used for the color maps, folder ‘image-URLs’ contains all image URLs for ProteinAtlas images and folder ‘sample_images’ contains image files for all remaining images. (ZIP) [file pone.0145572.s005.zip › S2_file/sample_images/Orig_Cell_Pos_X1256-Y3175-W65-H69.png]

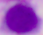

Supplement: S2 File — This ZIP file contains all raw data used for the experiments. Folder ‘data’ contains a list of colors used for the color maps, folder ‘image-URLs’ contains all image URLs for ProteinAtlas images and folder ‘sample_images’ contains image files for all remaining images. (ZIP) [file pone.0145572.s005.zip › S2_file/sample_images/Orig_Cell_Pos_X1920-Y335-W43-H35.png]

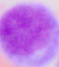

Supplement: S2 File — This ZIP file contains all raw data used for the experiments. Folder ‘data’ contains a list of colors used for the color maps, folder ‘image-URLs’ contains all image URLs for ProteinAtlas images and folder ‘sample_images’ contains image files for all remaining images. (ZIP) [file pone.0145572.s005.zip › S2_file/sample_images/Orig_Cell_Pos_X310-Y425-W59-H67.png]

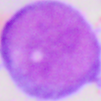

Supplement: S2 File — This ZIP file contains all raw data used for the experiments. Folder ‘data’ contains a list of colors used for the color maps, folder ‘image-URLs’ contains all image URLs for ProteinAtlas images and folder ‘sample_images’ contains image files for all remaining images. (ZIP) [file pone.0145572.s005.zip › S2_file/sample_images/Orig_Cell_Pos_X361-Y137-W101-H101.png]

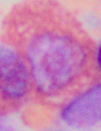

Supplement: S2 File — This ZIP file contains all raw data used for the experiments. Folder ‘data’ contains a list of colors used for the color maps, folder ‘image-URLs’ contains all image URLs for ProteinAtlas images and folder ‘sample_images’ contains image files for all remaining images. (ZIP) [file pone.0145572.s005.zip › S2_file/sample_images/Orig_Cell_Pos_X607-Y2792-W101-H131.png]

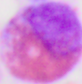

Supplement: S2 File — This ZIP file contains all raw data used for the experiments. Folder ‘data’ contains a list of colors used for the color maps, folder ‘image-URLs’ contains all image URLs for ProteinAtlas images and folder ‘sample_images’ contains image files for all remaining images. (ZIP) [file pone.0145572.s005.zip › S2_file/sample_images/Orig_Cell_Pos_X747-Y119-W82-H84.png]

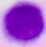

Supplement: S2 File — This ZIP file contains all raw data used for the experiments. Folder ‘data’ contains a list of colors used for the color maps, folder ‘image-URLs’ contains all image URLs for ProteinAtlas images and folder ‘sample_images’ contains image files for all remaining images. (ZIP) [file pone.0145572.s005.zip › S2_file/sample_images/Orig_Cell_Pos_X853-Y6-W46-H47.png]

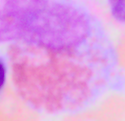

Supplement: S2 File — This ZIP file contains all raw data used for the experiments. Folder ‘data’ contains a list of colors used for the color maps, folder ‘image-URLs’ contains all image URLs for ProteinAtlas images and folder ‘sample_images’ contains image files for all remaining images. (ZIP) [file pone.0145572.s005.zip › S2_file/sample_images/Orig_Cell_Pos_X980-Y416-W125-H121.png]

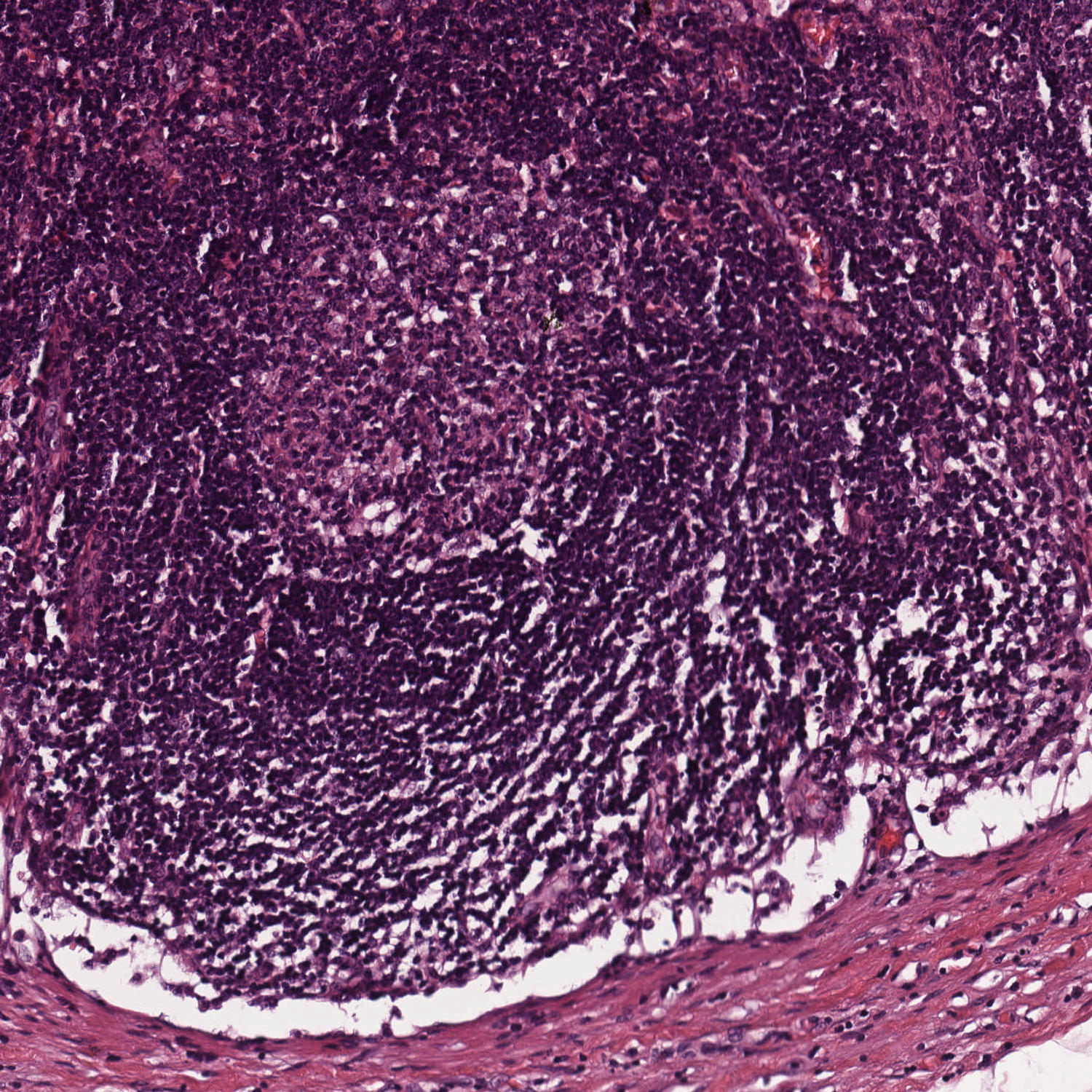

Supplement: S2 File — This ZIP file contains all raw data used for the experiments. Folder ‘data’ contains a list of colors used for the color maps, folder ‘image-URLs’ contains all image URLs for ProteinAtlas images and folder ‘sample_images’ contains image files for all remaining images. (ZIP) [file pone.0145572.s005.zip › S2_file/sample_images/Sample001_1500px.tiff]

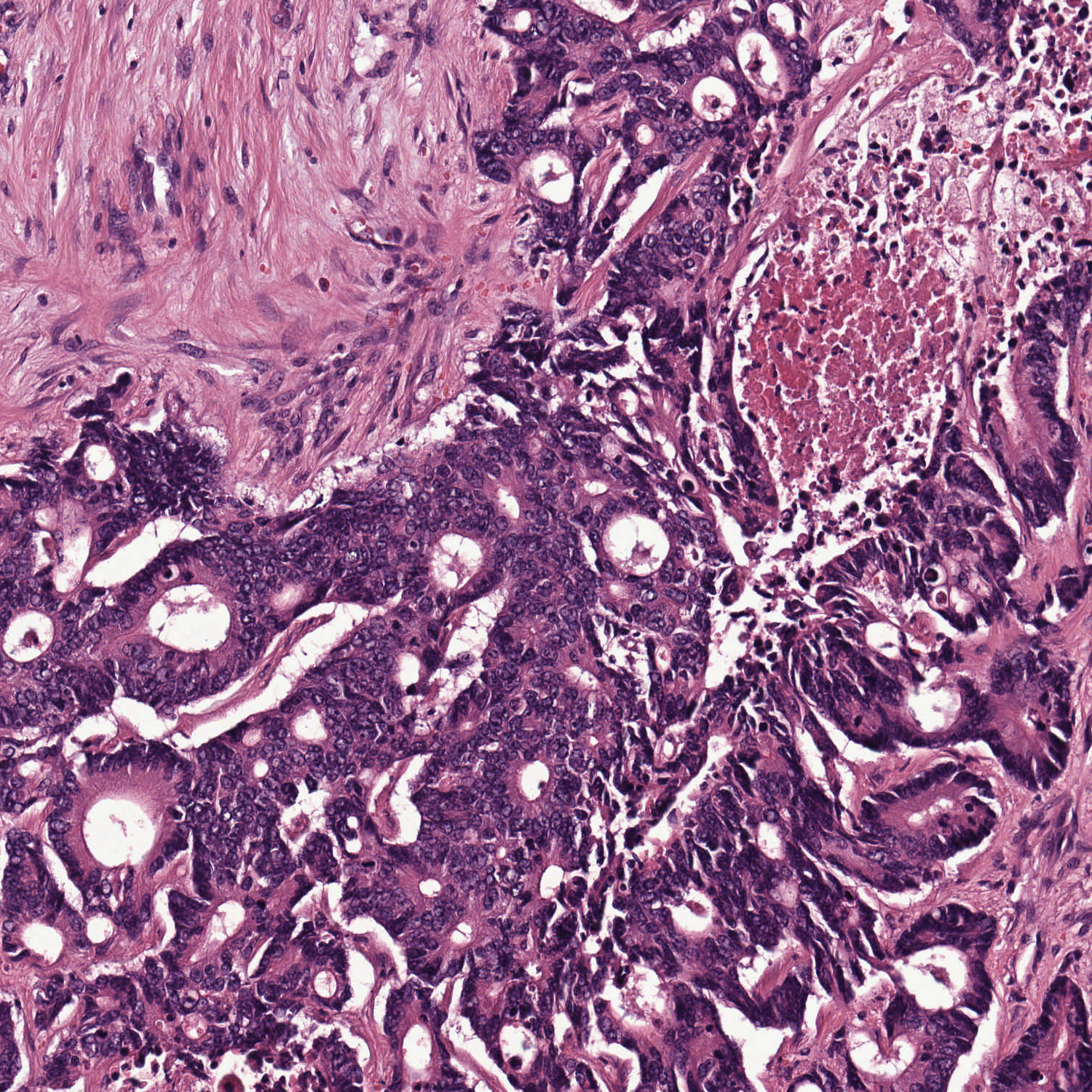

Supplement: S2 File — This ZIP file contains all raw data used for the experiments. Folder ‘data’ contains a list of colors used for the color maps, folder ‘image-URLs’ contains all image URLs for ProteinAtlas images and folder ‘sample_images’ contains image files for all remaining images. (ZIP) [file pone.0145572.s005.zip › S2_file/sample_images/Sample002_1500px.tiff]

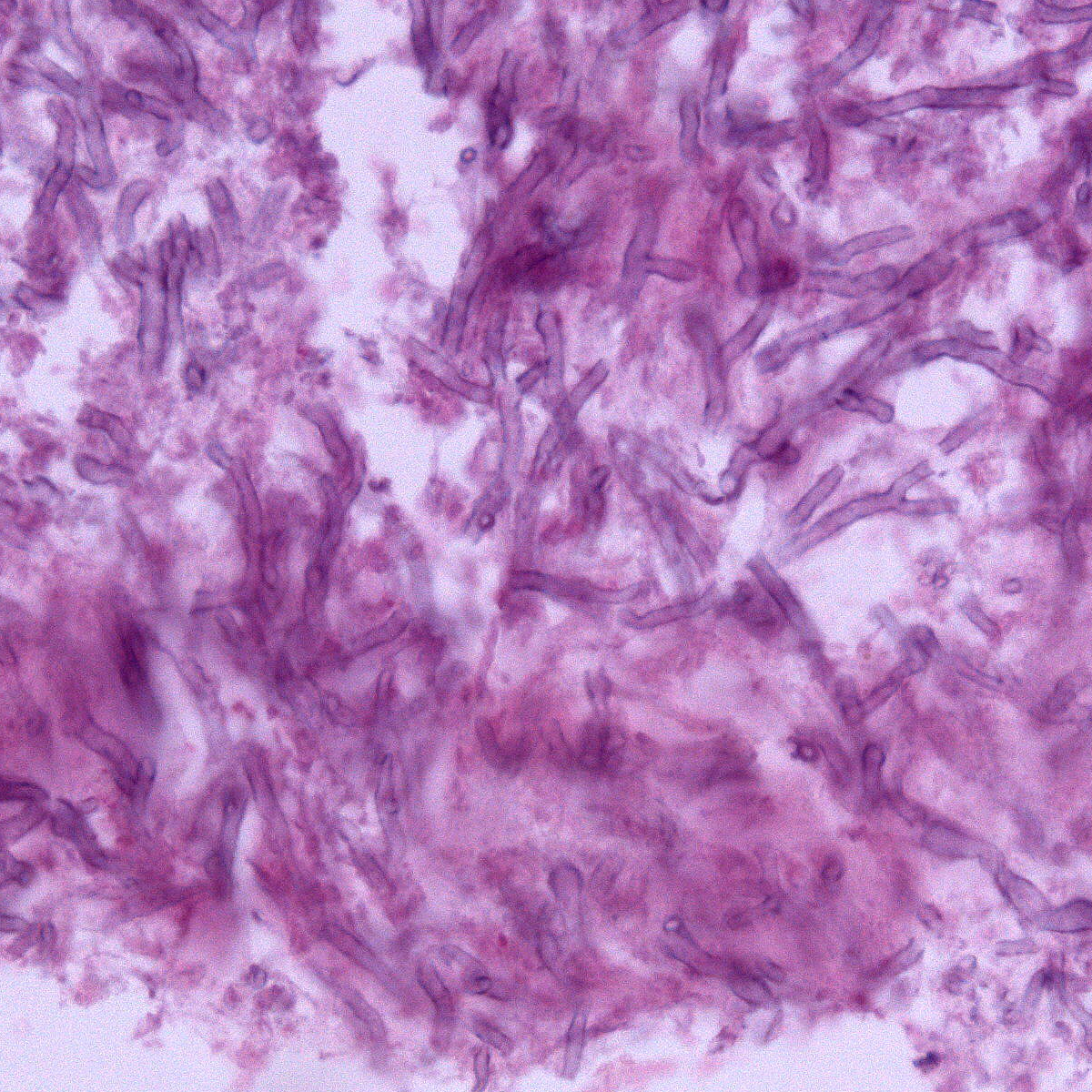

Supplement: S2 File — This ZIP file contains all raw data used for the experiments. Folder ‘data’ contains a list of colors used for the color maps, folder ‘image-URLs’ contains all image URLs for ProteinAtlas images and folder ‘sample_images’ contains image files for all remaining images. (ZIP) [file pone.0145572.s005.zip › S2_file/sample_images/Sample003_1200px.tiff]

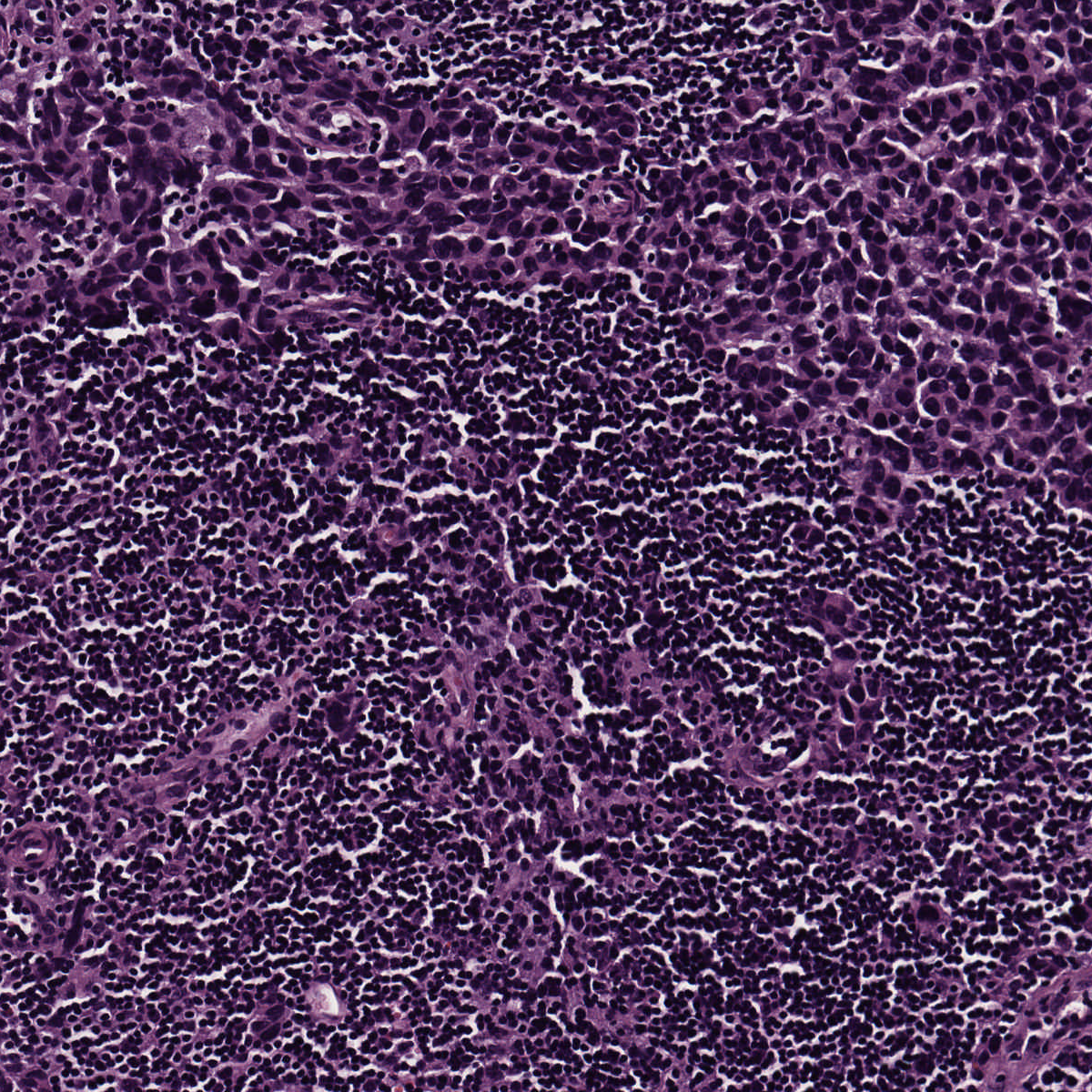

Supplement: S2 File — This ZIP file contains all raw data used for the experiments. Folder ‘data’ contains a list of colors used for the color maps, folder ‘image-URLs’ contains all image URLs for ProteinAtlas images and folder ‘sample_images’ contains image files for all remaining images. (ZIP) [file pone.0145572.s005.zip › S2_file/sample_images/Sample004_1200px.tiff]

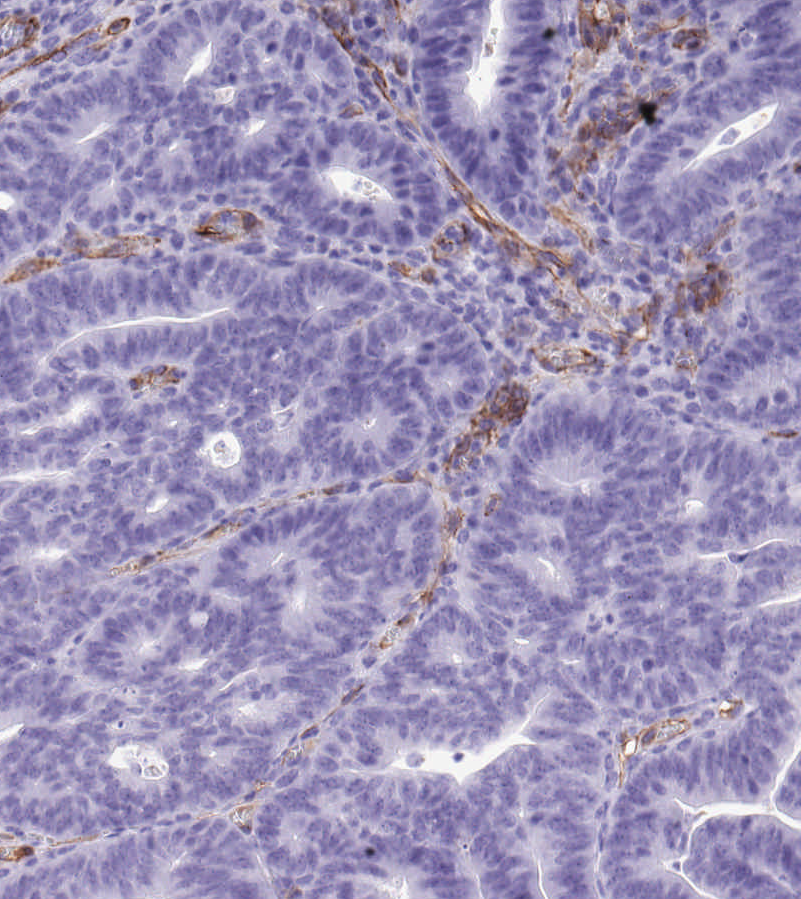

Supplement: S2 File — This ZIP file contains all raw data used for the experiments. Folder ‘data’ contains a list of colors used for the color maps, folder ‘image-URLs’ contains all image URLs for ProteinAtlas images and folder ‘sample_images’ contains image files for all remaining images. (ZIP) [file pone.0145572.s005.zip › S2_file/sample_images/Tumor_CD31_HiRes.png]

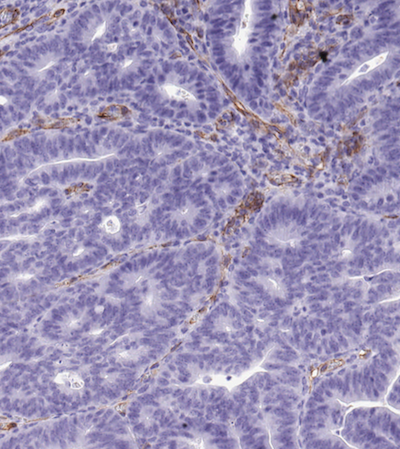

Supplement: S2 File — This ZIP file contains all raw data used for the experiments. Folder ‘data’ contains a list of colors used for the color maps, folder ‘image-URLs’ contains all image URLs for ProteinAtlas images and folder ‘sample_images’ contains image files for all remaining images. (ZIP) [file pone.0145572.s005.zip › S2_file/sample_images/Tumor_CD31_LoRes.png]
