# Supplementary material for: New Colors for Histology: Optimized Bivariate Color Maps Increase Perceptual Contrast in Histological Images
Source: PLoS One. 2015 Dec 30;10(12):e0145572. doi: 10.1371/journal.pone.0145572 (PMC4696851; doi:10.1371/journal.pone.0145572)
Supplement: S1 File — Table A: Contrast metrics for original and new color maps. Table B: Perceptual contrast improvement of objects in histological images. List A: Details of the computational procedures. List B: Image credit. (PDF) [file pone.0145572.s004.pdf]

S1 File

Table A: Contrast metrics for original and new color maps.

color map	hex	max contrast	mean contrast	std. dev.
red – blue 1	#FF0000-#0093FF	88.1	35.9	15.1
red – blue 2	#E12839-#0081FF	65.8	31.8	12.6
blue – orange 1	#008AFF-#FD6500	79.7	34.9	14.3
blue – orange 2	#0077FF-#FF8E00	79.6	36.1	15
blue – orange 3	#006EFF-#FFAD00	82.6	37.4	16
brown – blue *	#B58C70-#5C5FA1	51.7	22.9	10.1

* original colors of H-DAB images. Units of contrast measurement are arbitrary.

Table B: Perceptual contrast improvement of objects in histological images.

dataset	N images	contrast before	contrast after	improvement	p-value
AFP-liver	85	15.5 +/- 5.8	33.7 +/- 7.8	116.99%	1.1819e-37
BRAF-mela	41	21 +/- 6.8	37.6 +/- 7	78.7%	2.0774e-21
ERBB2-brst	81	20.3 +/- 12.8	37.1 +/- 9.7	82.22%	6.2725e-30
ESR1-brst	80	35.2 +/- 14.6	46 +/- 11.6	30.66%	6.0519e-16
MKI67-crc	81	23.9 +/- 9.9	38.5 +/- 12.4	60.69%	7.5639e-33
MKI67-uro	83	24.7 +/- 11.2	40.3 +/- 12.4	63.3%	2.798e-37
PGR-brst	100	23.4 +/- 11.9	38 +/- 8.7	62%	1.0106e-35
S100A1-ova	45	18.3 +/- 9.3	35.3 +/- 8.4	92.71%	4.9013e-22

Contrast before and after the procedure is shown in arbitrary units (mean $\pm$ standard deviation).

List A: Details of the computational procedures.

In the MATLAB program for re-staining of H-DAB images, the following parameters were used:

```
nBinsCoarse = 18; % measure colors in each channel in 18 levels
nBinsFine = 60;   % use 60 color levels in each channel for output
resizeDeconvAnalyis = 0.2; % for estimating optimal color deconvolution
interpolType = 'linear'; % color map interpolation in 3D
convType = 'optimal'; % method for color deconvolution
sets = {'AFP-liver','ERBB2-brst','MKI67-crc',...
'PGR-brst','BRAF-mela', 'ESR1-brst', ...
'MKI67-uro','S100A1-ova'}; % data sets for analysis
```

In the MATLAB program for re-staining of H&E and Giemsa stained samples, the following parameters were used:

```
nBinsCoarse = 40; % measure colors in each channel in 30 levels
nBinsFine = 40;   % use 30 color levels in each channel for output
interpolType = 'linear'; % color map interpolation in 3D
```

For our experiments, we used a standard computer (2.2 GHz Intel Core i7, 16 GB DDR3 RAM, High-resolution display with Adobe RGB 1998 color profile). All codes were implemented in MATLAB and were not explicitly optimized for computational performance. Computational performance was mostly dependent on the color depth (binning) of the optimized color map. To re-stain an H&E image of $1500 * 1500$ px took between 2.6 sec (for a 10 x 10 color map) and 39.47 sec (for a 40 x 40 color map).

For all experiments, we used the CIE standard illuminant D65 as white point (RGB: 0.9504, 1.0000, 1.0888; color temperature 6504 K).

List B: Image credit.

The image shown in Fig. 8I and J was cropped from the following image on wikimedia commons (Creative Commons license):

https://commons.wikimedia.org/wiki/
File:HE_aspergillus_intraoperative_specimen.jpg
